# TGF-β/SMAD4/14-3-3σ/TFEB axis promotes mesenchymal-epithelial transition and inhibits autophagy in colorectal cancer

**DOI:** 10.1038/s41419-026-08733-x

**Published:** 2026-04-21

**Authors:** Xiaoyan Chen, Markus Winter, Matjaz Rokavec, Janine König, Heiko Hermeking

**Affiliations:** 1 Experimental and Molecular Pathology, Institute of Pathology, Ludwig-Maximilians-University München, Thalkirchner Strasse 36, D-80337 Munich, Germany; 2 German Cancer Consortium (DKTK), Partner Site Munich, D-80336 Munich, Germany; 3 German Cancer Research Center (DKFZ), D-69210 Heidelberg, Germany

**Keywords:** Colon cancer, Oncogenesis

## Abstract

*14-3-3σ* is a p53-inducible gene with tumor suppressive properties and SMAD4 is a transcription factor encoded by a tumor suppressor gene, which is commonly inactivated in colorectal cancer (CRC). Here, *14-3-3σ* was characterized as direct transcriptional target of SMAD4. TGF-β treatment of tumoroids derived from CRC patients and mouse models resulted in a *SMAD4*-dependent induction of *14-3-3σ*. In murine, intestinal epithelia, the apical expression of *14-3-3σ* was dependent on *Smad4*. Ectopic SMAD4 or 14-3-3σ promoted mesenchymal-to-epithelial transition (MET) and suppressed invasion, migration, and autophagy of CRC cells. As experimental inactivation of 14-3-3σ abolished these tumor-suppressive functions of SMAD4, 14-3-3σ mediates these effects of SMAD4. Inhibition of autophagy and promotion of MET by SMAD4 was mediated by inhibition of TFEB via binding and sequestration of TFEB by 14-3-3σ. The association of 14-3-3σ and TFEB was dependent on phosphorylation of the TFEB serine 211 residue, which is a target of mTORC1. Taken together, the TGF-β/SMAD4/14-3-3σ/TFEB axes characterized here antagonizes epithelial plasticity and autophagy. Thereby, it may ultimately suppress the progression of CRC and other types of cancer.

## Introduction

Colorectal cancer (CRC), the third most prevalent cancer type world-wide, leads to the second highest number of cancer-related deaths with 1.9 million new cases and 935,000 deaths each year [1, 2]. Despite a decrease in the general incidence and mortality rates, CRC is being detected more frequently in younger individuals and at more advanced stages and tends to locate mostly in the left colon and rectum [3]. The considerable mortality associated with CRC is largely due to its metastatic potential. Even with treatment advances, the five-year survival rate of patients with metastatic CRC (mCRC) remained at approximately 14% [1, 4]. Therefore, gaining deeper insights into the molecular pathways governing CRC metastasis is fundamental to improving mCRC prevention and treatment.

The tumor suppressor gene *SMAD4*/*DPC4* maps to human chromosome 18q21 [5, 6]. Loss of chromosome 18q occurs in more than 60% of colorectal and other cancer types [5, 7]. Notably, loss of *SMAD4* accounts for ~60% of the 18q21.1 allelic losses in CRC [8]. The SMAD4 transcription factor is a central mediator of the canonical transforming growth factor-beta (TGF-β) pathway [9, 10]. When TGF-β binds to its receptor, receptor-bound SMADs (R-SMADs) are phosphorylated, activated and form a complex with SMAD4 which translocates to the nucleus, and binds to SMAD-binding elements (SBEs) in the promoters or enhancers of target genes [9]. Moreover, SMAD4 associates with other DNA-binding proteins and co-factors to regulate genes involved in cell growth, proliferation, differentiation, apoptosis, and matrix production [11].

EMT and the reverse process, mesenchymal-epithelial transition (MET), facilitate cellular invasion and migration, and ultimately metastasis formation. EMT promotes the spread of cancer cells and MET is necessary for their colonization at secondary sites [12]. SMAD4 is a key tumor suppressor that can mediate anti-metastatic effects of TGF-β signaling in CRC by limiting migration and invasion [13]. The frequency of *SMAD4* gene mutations is elevated in CRC patients with distant metastases, particularly in liver metastases, and is associated with poor prognosis [14, 15]. Furthermore, patients with CRCs expressing high SMAD4 levels display significantly improved overall and disease-free survival when compared to patients with low SMAD4 levels in CRCs [16]. The clinical significance of *SMAD4* inactivation is not limited to CRC, e.g. in pancreatic ductal adenocarcinoma it is associated with metastatic dissemination and poor patient prognosis [17]. Concomitant deletion of *Smad4* and *Apc* synergistically accelerates intestinal tumorigenesis and progression when compared to loss of *Apc* alone, indicating that loss of Smad4 function promotes the advancement and metastatic potential of CRC in vivo [18].

The 14-3-3σ protein (also known as Stratifin/SFN) belongs to the highly conserved 14-3-3 family and has been implicated in cell cycle control, apoptosis, and signal transduction [19]. In contrast to other 14-3-3 family members, 14-3-3σ exclusively forms homodimers [19]. This dimerization is important as it creates a pocket that mediates binding to ligand proteins via a motif with phosphorylated serine/threonine residues [20]. *14-3-3σ* is directly induced by the p53 tumor suppressor after its activation by DNA damage [21]. 14-3-3σ sequesters cell cycle regulators, such as the CDC2-cyclin B1 complex, in the cytoplasm, resulting in a G_2_/M arrest [21]. *14-3-3σ* is commonly down-regulated in cancers after *p53* inactivation and by epigenetic silencing due to CpG methylation [22–24]. Deletion of *14-3-3σ* significantly increases intestinal tumor formation in *Apc*^*Min/+*^ mice and shortens their life-span, suggesting a critical tumor-suppressive role of *14-3-3σ* in CRC [25].

TFEB, member of the MiT/TFE family of basic helix-loop-helix-leucine-zipper (bHLH-Zip) transcription factors, is a master regulator of lysosomal biogenesis and autophagy [26]. Nuclear TFEB promotes transcription of genes within the coordinated lysosomal expression and regulation (CLEAR) motif, driving lysosomal function and cellular clearance [27]. Accelerated tumorigenesis is characterized by high energy consumption and scarcity of biosynthetic precursors relative to normal cells [28]. Thus, increased autophagolysosomal flux is a common feature of tumors, and makes TFEB an attractive subject in the study of cancer. Interestingly, TFEB is sequestered in the cytoplasm through phosphorylation and binding to 14-3-3 proteins (such as 14-3-3α/β and γ) [29]. However, it is unknown whether 14-3-3σ can bind to TFEB and block its function by cytoplasmic sequestration.

We had previously shown that *14-3-3σ* expression increases apically in villi of intestinal epithelia [25]. As TGF-β signaling also increases in a similar pattern, we hypothesized that 14-3-3σ may represent a direct target and effector of the TGF-β pathway. We found that TGF-β induces *14-3-3σ* via activating SMAD4, which directly binds to the *14-3-3σ* promoter. Furthermore, SMAD4 promoted MET and inhibited autophagy through 14-3-3σ, which retained the transcription factor TFEB in the cytoplasm and thereby inhibited autophagy. Taken together, this pathway is likely to represent an important mediator of the suppressive effect of SMAD4 on CRC invasion and metastasis.

## Results

### *14-3-3σ* is a direct target gene of SMAD4 in human CRC cells

**Fig. 1:**
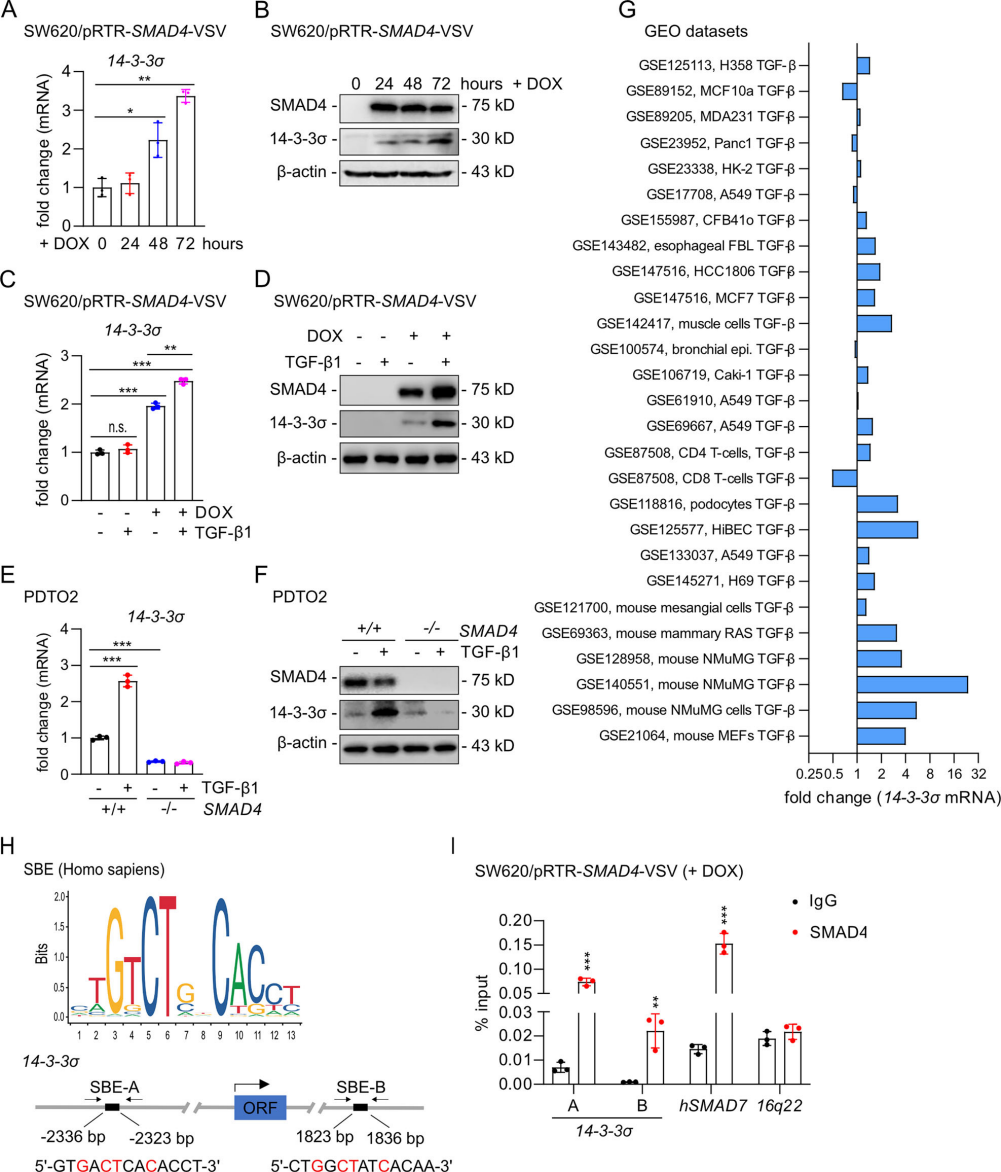
*14-3-3σ* is a direct target gene of SMAD4 in human CRC cells. **A** qPCR analysis of *14-3-3σ* expression in SW620 cells after SMAD4 induction with DOX for the indicated periods. **B** Western blot analysis of 14-3-3σ in SW620 cells treated as in **A**. **C** qPCR analysis of *14-3-3σ* expression in SW620 cells after treatment with DOX and/or 20 ng/mL recombinant TGF-β1 for 48 h. **D** Western blot analysis of 14-3-3σ in SW620 cells treated as in **C**. **E** qPCR analysis of *14-3-3σ* expression in *SMAD4* wild-type and *SMAD4* knockout PDTO2 after treatment with/without 20 ng/mL recombinant TGF-β1 for 72 h. **F** Western blot analysis of 14-3-3σ in PDTO2 cells treated as in **E**. **G** Expression changes of *14-3-3σ* in response to TGF-β1 stimulations across multiple GEO datasets. Bars represent fold change values of *14-3-3σ* relative to control, with datasets from both human and mouse cell types indicated on the y-axis. **H** The top panel shows SMAD4 potential binding site in jaspar.genereg.net. The bottom panel shows the map of human *14-3-3σ* genomic regions with indicated SMAD4 binding sites. **I** qChIP analysis of SMAD4 occupancy at the human *14-3-3σ* genomic regions in SW620 cells treated with DOX for 48 h. Human *SMAD7* and *16q22* served as positive and negative controls, respectively. Results are presented as the mean ± SD (*n* = 3) for panel **A**, **C**, **E** and **I** with *p < 0.05, **p < 0.01, ***p < 0.001.

In order to determine, whether 14-3-3σ may represent an effector of the TGF-β pathway, we first determined whether SMAD4 induces the expression of *14-3-3σ*. Therefore, SMAD4 was ectopically expressed in the colorectal cancer cell line SW620, which is known to be *SMAD4*-deficient [8], using the conditional, doxycycline (DOX)-inducible pRTR vector system described before [30]. As expected, treatment with doxycycline (DOX) resulted in a time-dependent increase in *14-3-3σ* mRNA and 14-3-3σ protein expression (Fig. 1A, B). TGF-β1 treatment further enhanced SMAD4-induced 14-3-3σ mRNA and protein levels in SW620 cells (Fig. 1C, D), presumably by inducing the phosphorylation of SMAD. Similar results were obtained in SW480 cells (Fig. S1A-D). Furthermore, deletion of *SMAD4* in a patient-derived tumor organoid (PDTO) abolished the TGF-β1-induced up-regulation of 14-3-3σ expression (Fig. 1E, F). Similar results were obtained in another PDTO (Fig. S1E). These results imply that the induction of 14-3-3σ by TGF-β is mediated by SMAD4. To evaluate the regulatory effect of TGF-β on *14-3-3σ* expression, we performed an integrative analysis of GEO transcriptomic datasets (Fig. 1G). Overall, TGF-β stimulation resulted in a consistent induction of *14-3-3σ* in different cell types and species. Of note, TGF-β induced an elevated expression of *14-3-3σ* in several mouse cell models. In the promoter region of *14-3-3σ* we identified several potential SMAD binding sites (SBE) using the JASPAR algorithm (Fig. 1H). qChIP analysis confirmed that SMAD4 occupies two predicted SBEs up- and down-stream of the *14-3-3σ* coding region (Fig. 1H, I). Taken together, these results demonstrate that human *14-3-3σ* is a direct target gene of SMAD4.

### Murine *14-3-3σ* is a direct target gene of Smad4

**Fig. 2:**
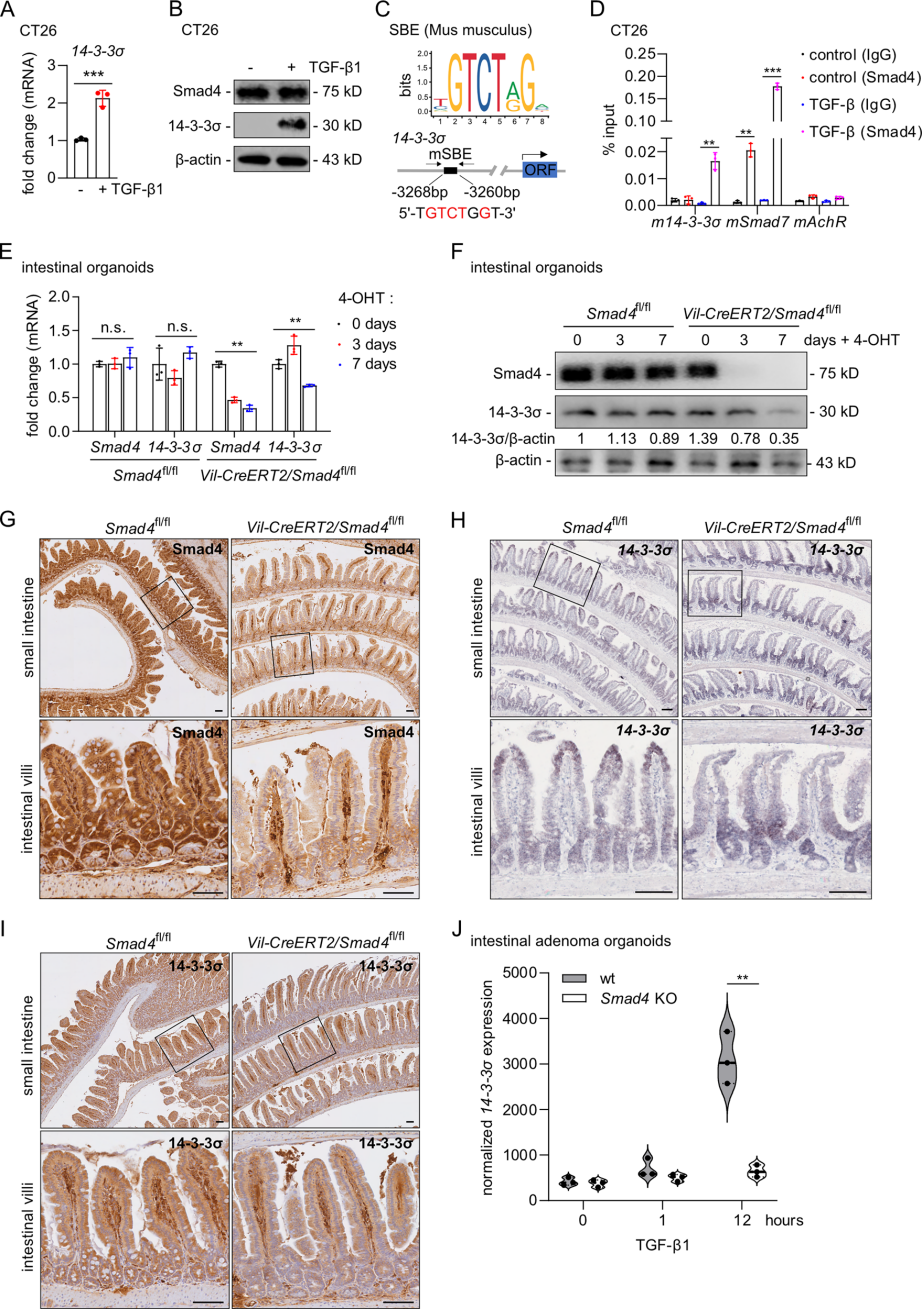
*14-3-3σ* is a direct target gene of Smad4 in murine CRC cells and mice. **A** PCR analysis of *14-3-3σ* in CT26 cells after treatment with TGF-β1 for 72 h. **B** Western blot analysis of 14-3-3σ in CT26 cells after treatment with TGF-β1 for 72 h. **C** The top panel shows that the map of murine *14-3-3σ* genomic regions with indicated Smad4 binding sites. The bottom panel is the map of mouse *14-3-3σ* genomic regions with indicated Smad4 binding sites. **D** qChIP analysis of Smad4 occupancy at the murine *14-3-3σ* genomic regions in CT26 after treatment with/without 20 ng/mL recombinant TGF-β1 for 48 h. Murine *Smad7* and *16q22* served as positive and negative controls, respectively. **E** qPCR analysis of *Smad4* and *14-3-3σ* expression in organoids isolated from three male mice of each genotype treated with 4-OHT for the indicated days. **F** Western blot analysis of indicated protein levels in organoids treated as in **E**. **G** Immunohistochemical detection of Smad4 in 8 weeks old mice of the indicated genotype. *n* = 3 mice per genotype. Scale bar represent 100 μm. **H** *14-3-3σ* mRNA was detected by ISH in 8 weeks old mice of the indicated genotype. *n* = 3 mice per genotype. Scale bar represents 100 μm. **I** Immunohistochemical detection of 14-3-3σ in 8 weeks old mice of the indicated genotype. *n* = 3 mice per genotype. Scale bar represents 100 μm. **J** Comparison of *14-3-3σ* expression in *Apc*^-/-^/*Smad4*^*+/+*^ and *Apc*^*-/-*^*/Smad4*^*-/-*^ organoids after TGF-β1 exposure for the indicated times. In panel A, D, E, and J mean values ± SD are shown (*n* = 3). **p < 0.01, ***p < 0.001. n.s. no significance.

Next we determined, whether the regulation of *14-3-3σ* by Smad4 is conserved between species. When the murine colorectal cell line CT26 was treated with TGF-β1 an increase in 14-3-3σ mRNA and protein levels was observed (Fig. 2A, B). Bioinformatics analysis hinted to a potential SBE approximately 3 kbp upstream of the murine *14-3-3σ* promoter (Fig. 2C). qChIP analysis confirmed that Smad4 directly binds this SBE (Fig. 2C, D). To investigate whether Smad4 regulates *14-3-3σ* expression in the mouse intestine, we generated *Villin-CreERT2/Smad4*^*fl/fl*^ mice, which allow the conditional, intestinal epithelial cell-specific deletion of *Smad4*. From these mice we derived intestinal organoids. After exposure to 4-hydroxy-tamoxifen/4-OHT the expression of 14-3-3σ was significantly reduced on the mRNA and protein levels (Fig. 2E, F). Furthermore, *14-3-3*σ expression was analysed in 8-week-old mice after intraperitoneal (IP) injection of Tamoxifen to induce deletion of *Smad4* in vivo (Fig. S2A). Immunohistochemical analysis confirmed the absence of Smad4 expression in the small intestinal epithelium when compared to control *Smad4*^*fl/fl*^ mice (Fig. 2G). Similar reductions were observed in the colonic epithelium (Fig. S2B). Deletion of *Smad4* resulted in a lack of increased 14-3-3σ expression at mRNA and protein levels in the apical regions of epithelia in the small intestine (Fig. 2H, I) and the colon (Fig. S2C, D). To further explore the transcriptional response induced by TGF-β1, we re-analyzed the RNA-seq dataset published by Surakhy et al. (2025), which characterized the expression of *Smad4*-wildtype and *Smad4*-knockout adenoma organoids after 0, 1, and 12 h of TGF-β1 treatment [31]: *14-3-3σ* expression was significantly up-regulated in *Smad4* wild-type organoids after 12 h of TGF-β1 treatment, whereas no significant induction was observed in *Smad4*-deficient organoids (Fig. 2J). These results demonstrate that murine *14-3-3σ* is a direct target gene of Smad4 and that intestinal epithelia display *Smad4*-dependent expression of 14-3-3σ during differentiation.

### Functional effects of SMAD4-induced MET

**Fig. 3:**
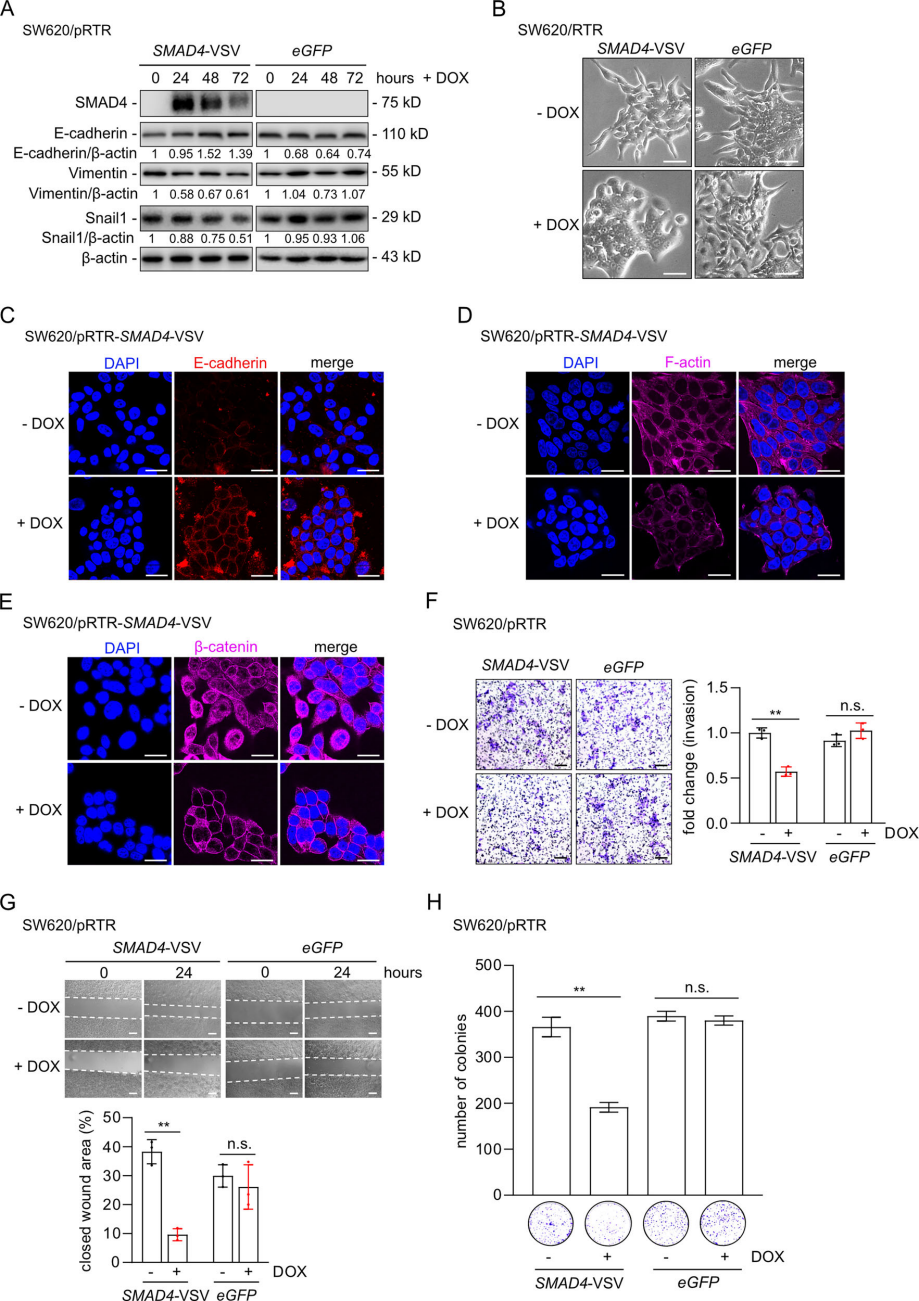
SMAD4 induced MET inhibits invasion, wound healing, and colony formation. **A** Western blot analysis of indicated proteins after SMAD4 induction by DOX in SW620 cells for the indicated periods. As a control, SW620 cells containing a pRTR vector were also treated with DOX for the indicated periods. **B** Representative phase contrast images show the morphology of SW620 cells with or without the addition of DOX. eGFP induced by the addition of DOX served as a control. Scale bar: 50 μm. **C–E** E-cadherin, F-actin and β-catenin were detected by immunofluorescence after SW620 cells were treated with or without DOX for 48 h. Nuclear DNA was stained with DAPI. Scale bar: 20 μm. **F** Invasion was measured using a modified Boyden chamber assay. After 48 h of DOX treatment, the number of cells that invaded through the Matrigel was counted by crystal violet staining. **G** Wound healing assay after 48 h of treatment with or without DOX. Width of the wound was determined 24 h after scratching (top). The results represent the mean (%) of wound healing (bottom). Scale bar: 50 μm. **H** Colony formation assay of the indicated cells treated with or without DOX for 48 h. In panel **F**–**H** mean values ± SD are shown (*n* = 3). **p < 0.01, n.s. no significance.

Ectopic expression of SMAD4 in SW620 CRC cells resulted in increased expression of the epithelial marker E-cadherin and decreased levels of the mesenchymal markers Vimentin and Snail1 (Fig. 3A). It also induced a morphology shift from spindle-shaped, mesenchymal-like cells to increased cell-cell contacts and loss of pseudopodia, indicating MET (ΔMET, Fig. 3B). In contrast, doxycycline (DOX) treatment of SW620 cells carrying an empty pRTR vector did not cause these changes in protein expression, nor morphological changes (Fig. 3A, B). Similar results were observed in SW480 cells upon ectopic SMAD4 expression (Fig. S3A, B). Furthermore, SMAD4 activation in SW620 cells resulted in a relocation of E-cadherin to the outer cell membrane, a marker specific for an epithelial state (Fig. 3C). Reorganization of the F-actin cytoskeletal and activation of β-catenin/TCF4 signaling are hallmarks of EMT/MET [30, 32, 33]. SMAD4 activation also induced rearrangement and reduction of cytoplasmic F-actin stress fibers (Fig. 3D). Additionally, SMAD4 re-expression induced relocation of β-catenin from the cytoplasm and nucleus to the plasma membrane, thereby presumably inhibiting β-catenin/TCF4 transcriptional activity (Fig. 3E). This is consistent with a previous report showing similar β-catenin re-localization in SW620 cells following ectopic SMAD4 expression [34]. Furthermore, ectopic SMAD4 expression significantly inhibited invasion, migration, and colony formation, while no inhibitory effects were observed in cells transfected with the empty pRTR vector (Fig. 3F–H). Similarly, activation of SMAD4 inhibited invasion and migration in SW480 cells (Fig. S3C, D). Therefore, ectopic SMAD4 induces MET and suppresses invasion, migration, and clonogenicity in mesenchymal-like CRC cell lines.

### Functional effects of 14-3-3σ-induced MET

**Fig. 4:**
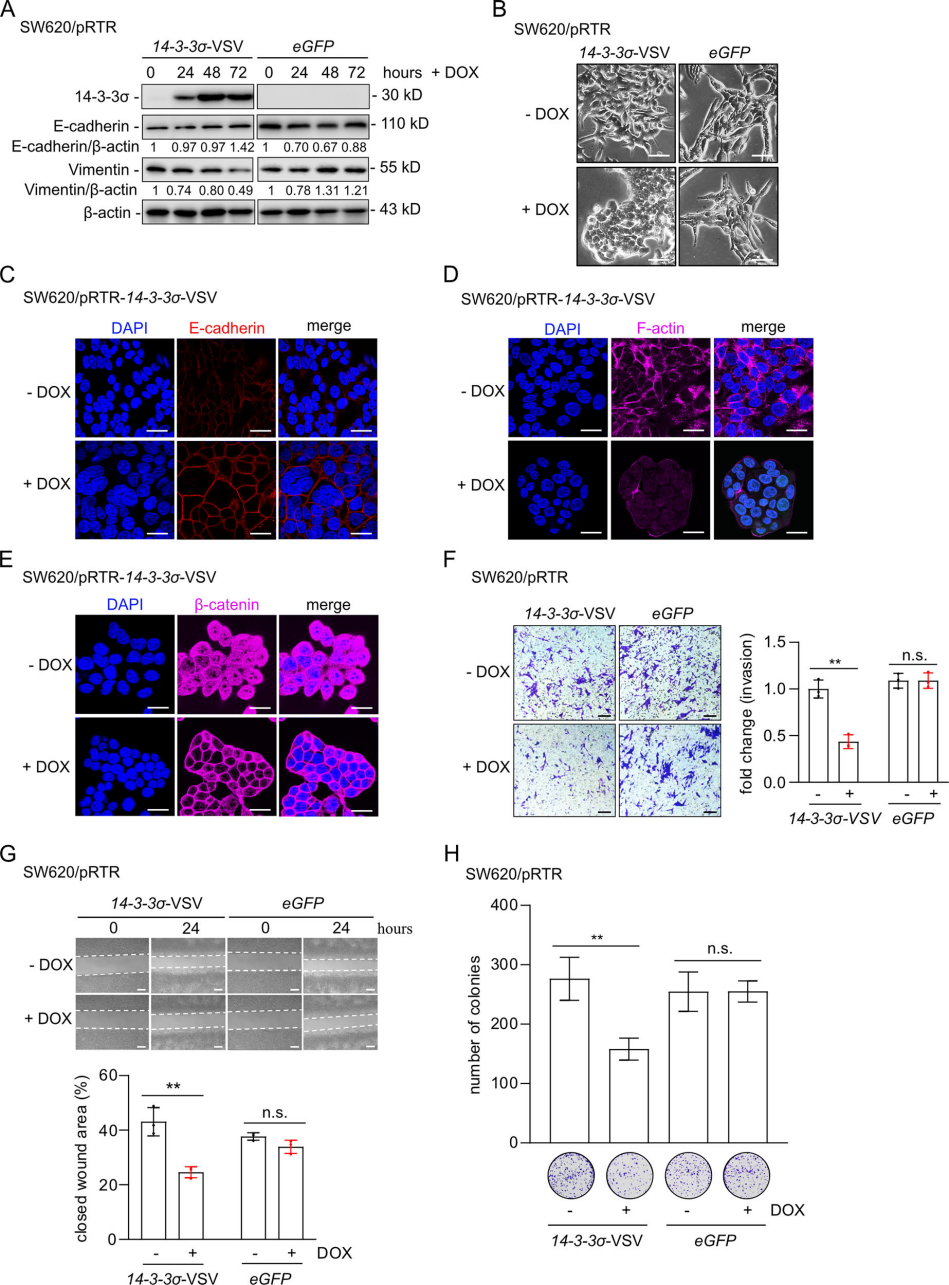
14-3-3σ induced MET inhibits invasion, wound healing, and colony formation. **A** Western blot analysis of indicated proteins after 14-3-3σ induction by DOX in SW620 cells for the indicated periods. As a control, SW620 cells containing a pRTR vector were also treated with DOX for the indicated periods. **B** Representative phase contrast images show the morphology of SW620 cells with or without the addition of DOX. eGFP induced with DOX served as a control. Scale bar: 50 μm. **C–E** E-cadherin, F-actin and β-catenin were detected by immunofluorescence after SW620 cells were treated with or without DOX for 48 h. Nuclear DNA was stained with DAPI. Scale bar: 20 μm. **F** Invasion was measured using a modified Boyden chamber assay. After 48 h of DOX treatment, the number of cells that invaded through the Matrigel was counted by crystal violet staining. **G** Wound healing assay after 48 h of treatment with or without DOX. Width of the wound was determined 24 h after scratching (top). The results represent the mean (%) of wound healing (bottom). Scale bar: 50 μm. **H** Colony formation assay of the indicated cells treated with or without DOX for 48 h. In **F**–**H** mean values ± SD are shown (*n* = 3). **p < 0.01, n.s. no significance.

Previous studies in adenomas from *Apc*^*Min/*+^ mice demonstrated that deletion of *14-3-3σ* leads to up-regulation of mesenchymal markers (Snail1, ZEB1, and Vimentin) and down-regulation of epithelial markers (CDH1, EPCAM) [25]. These results implied that *14-3-3σ* is required, at least to some extent, for keeping cells in an epithelial state. To determine whether 14-3-3σ is sufficient for inducing MET, we generated SW620 CRC cell pools with inducible, ectopic expression of *14-3-3σ*. Ectopic expression of 14-3-3σ led to increased E-cadherin and reduced Vimentin levels (Fig. 4A), and induced a morphological shift from a mesenchymal to an epithelial phenotype (Fig. 4B). No corresponding changes were observed in cells transfected with the empty pRTR vector. In addition, ectopic 14-3-3σ resulted in a localization of E-cadherin to the outer membrane, loss and re-organization cytoplasmic F-actin, and translocation of β-catenin from the cytoplasm and nucleus to the outer cell membrane (Fig. 4C–E). Furthermore, ectopic 14-3-3σ inhibited cell invasion, migration, and colony formation, whereas no inhibitory effects were observed in control cells harboring the pRTR vector (Fig. 4F–H). These results show that 14-3-3σ is sufficient to induce MET and suppresses migration and invasion of mesenchymal-like CRC cells.

### 14-3-3σ mediates induction of MET by SMAD4

**Fig. 5:**
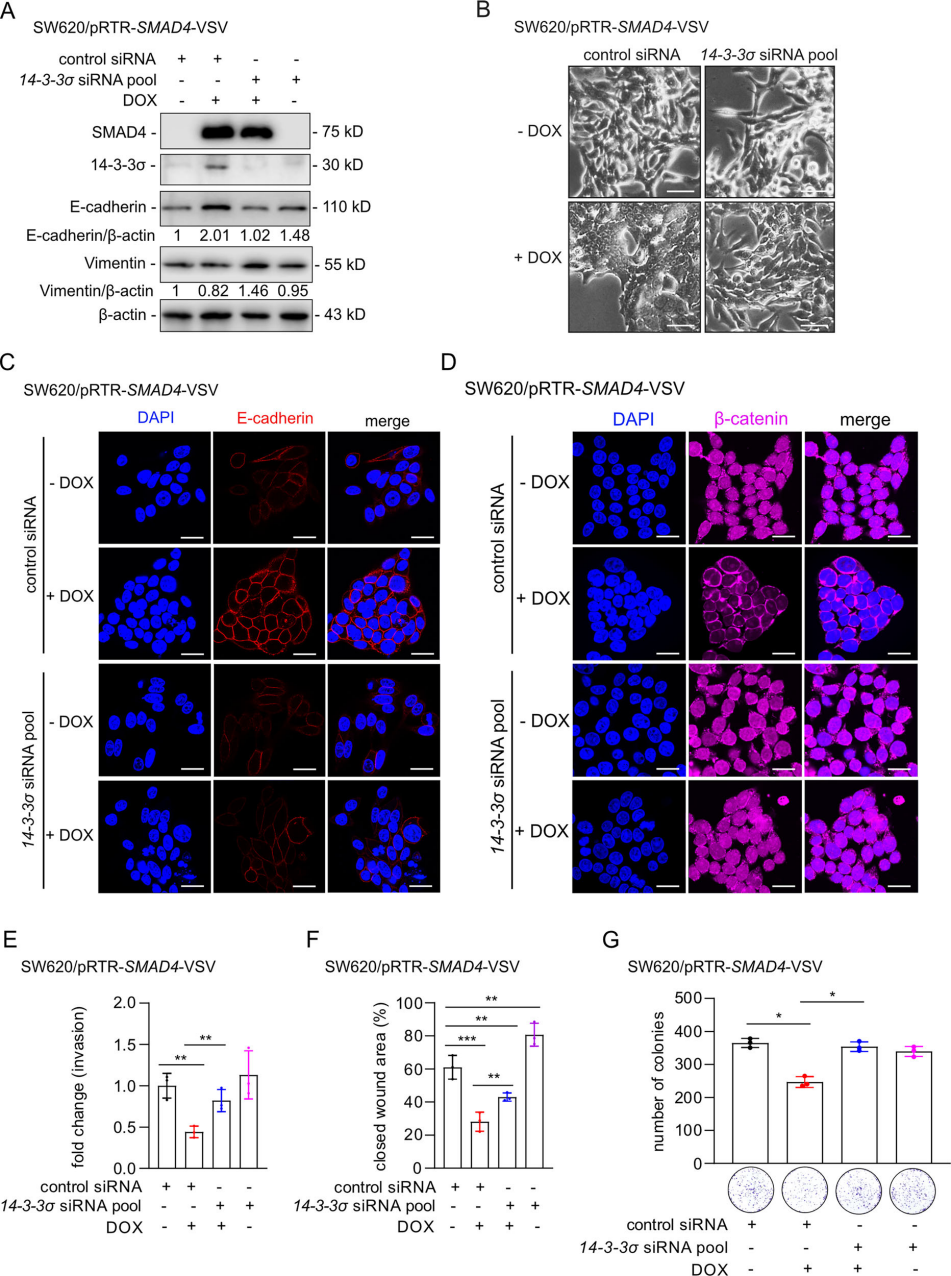
SMAD4 induced MET is mediated by 14-3-3σ. **A** Lysates were subjected to immunoblot analysis of the indicated proteins 72 h after induction of SMAD4 by addition of DOX and simultaneous transfection with the indicated siRNAs in SW620 cells. **B** Representative phase contrast images show the morphology of SW620 cells treated as in **A**. Scale bar: 50 μm. **C**, **D** Indirect immunofluorescence detection of E-cadherin and β-catenin after treatment as shown in **A**. Scale bar: 20 μm. **E** Boyden chamber analysis was used to quantify cell invasion after cells were treated as indicated. DOX was treated for 48 h. **F** Quantification of wound healing after the indicated treatments. **G** Quantification of colony formation after the indicated treatments. In **E**–**G** mean values ± SD are shown (*n* = 3). *p < 0.05, **p < 0.01, ***p < 0.001.

To further investigate the role of 14-3-3σ in SMAD4-induced MET, we concomitantly silenced 14-3-3σ using siRNA pools during activation of a conditional *SMAD4* allele (Fig. 5A). Ectopic SMAD4 increased E-cadherin expression, which was attenuated by silencing of *14-3-3σ*. Conversely, a SMAD4-mediated decrease in Vimentin was reversed following knock-down of *14-3-3σ*. Ectopic SMAD4 triggered MET, whereas cells with concomitant *14-3-3σ* silencing displayed a mesenchymal morphology (Fig. 5B). In addition, ectopic SMAD4 resulted in recruitment of E-cadherin to the membranes which was prevented by concomitant silencing of *14-3-3σ* (Fig. 5C). The SMAD4-induced redistribution and reduction of F-actin were dependent on 14-3-3σ, as simultaneous silencing of *14-3-3σ* abrogated these effects (Fig. S4A). Ectopic SMAD4 expression promoted β-catenin membrane localization, which was blocked by knock-down of *14-3-3σ* (Fig. 5D). *14-3-3σ* silencing partially reversed the SMAD4-induced suppression of cell invasion, migration, and colony formation (Fig. 5E–G, Fig. S4B, C). These findings demonstrate that 14-3-3σ mediates SMAD4-induced MET and the resulting inhibition of invasion and migration.

### 14-3-3σ mediates inhibition of autophagy by SMAD4

**Fig. 6:**
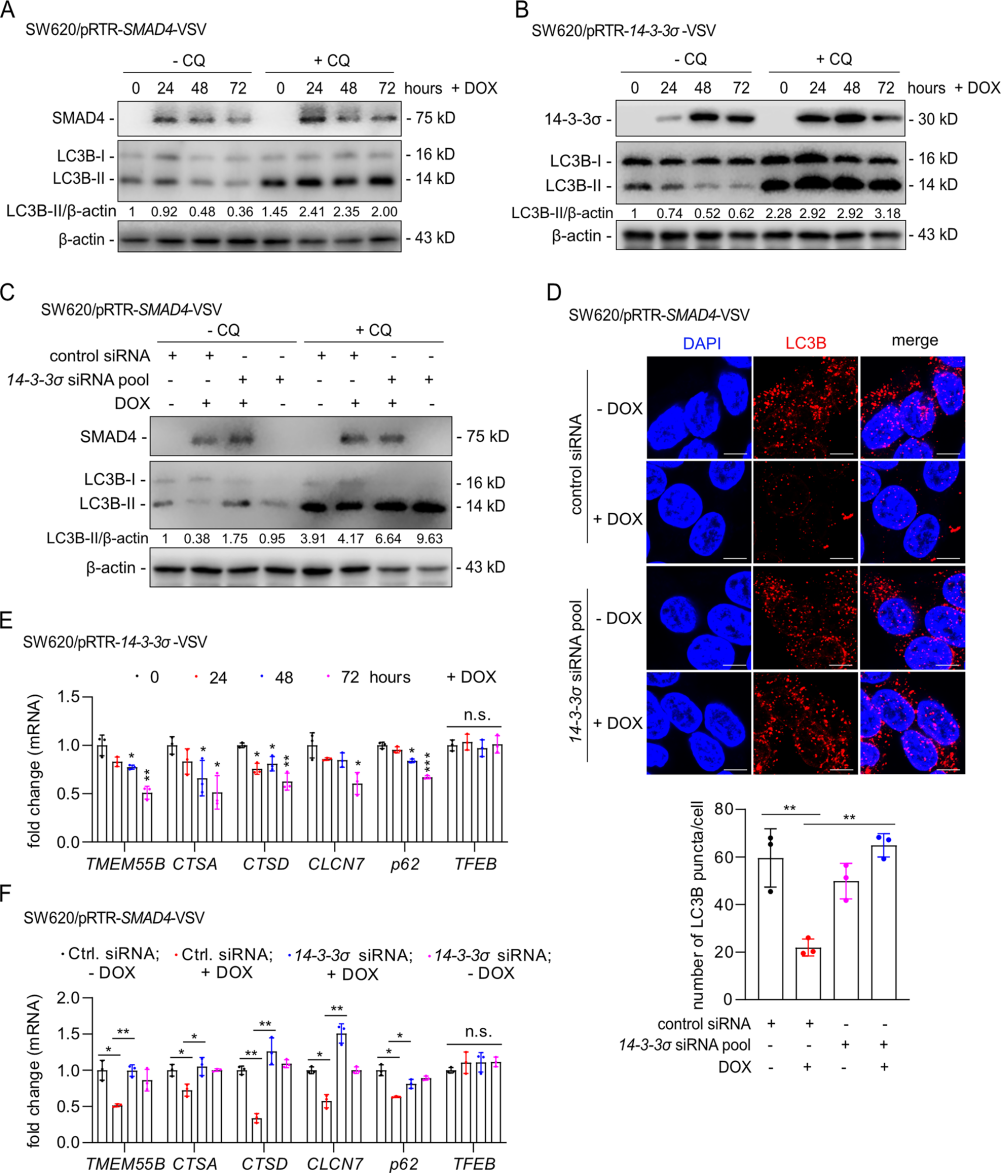
SMAD4 inhibits autophagy via inducing 14-3-3σ. **A** Immunoblot analysis of indicated protein levels in SW620 cells after induction of ectopic SMAD4 by addition of DOX for the indicated periods. 20 μM of chloroquine was added for the last 4 h before harvesting cells. **B** Immunoblot analysis of indicated protein levels after addition of DOX for the indicated periods to induce ectopic 14-3-3σ. 20 μM chloroquine was added for the last 4 h before harvesting cells. **C** SW620 cells were transfected with control siRNA or *14-3-3σ*-specific siRNA pools, and 24 h later, DOX was added for 48 h as indicated to induced ectopic SMAD4 expression. 20 μM chloroquine was added 4 h before harvesting cells. Immunoblot analysis of the indicated protein. **D** Indirect immunofluorescence detection of LC3B in SW620 cells transfected with the indicated siRNAs for 24 h and then treated with DOX for another 48 h. Nuclear DNA was stained with DAPI. Scale bar: 5 μm. The bar graph represents the quantification of puncta/cell. **E** qPCR analysis of the indicated mRNAs in SW620 cells treated with DOX for the indicated periods. **F** qPCR analysis of the indicated mRNAs after the treatments described in **D**. In **E**+**F** mean values ± SD are shown (*n* = 3). *p < 0.05, **p < 0.01, n.s. no significance.

Increased autophagy has been associated with a more mesenchymal stem cell-like phenotype and is required for invasion and migration of glioblastoma stem cell lines [35]. To investigate the regulatory roles of SMAD4 or 14-3-3σ in autophagy, we assessed the expression of the autophagy effector LC3B-II. Ectopic SMAD4 resulted decreased LC3B-II levels, indicating an inhibition of autophagy by SMAD4 (Fig. 6A). Similarly, ectopic 14-3-3σ decreased LC3B-II, and reduced cytoplasmic LC3B puncta (Fig. 6B, S5). Furthermore, silencing of 14-3-3σ abrogated the inhibitory effect of SMAD4 on autophagy (Fig. 6C). Moreover, ectopic SMAD4 expression reduced the number of cytoplasmic LC3B puncta, whereas concomitant silencing of *14-3-3σ* restored LC3B puncta formation in the presence of activated SMAD4 (Fig. 6D). Ectopic 14-3-3σ suppressed the autophagy-related genes *TMEM55B, CTSA, CTSD, CLC7* and *p62*, without affecting *TFEB* (Fig. 6E). Similarly, SMAD4 activation inhibited the same set of genes; however, this inhibitory effect was abolished upon simultaneous *14-3-3σ* silencing (Fig. 6F). Collectively, these results show that SMAD4 inhibits autophagy via inducing *14-3-3σ*.

### SMAD4 inhibits TFEB via inducing *14-3-3σ*

**Fig. 7:**
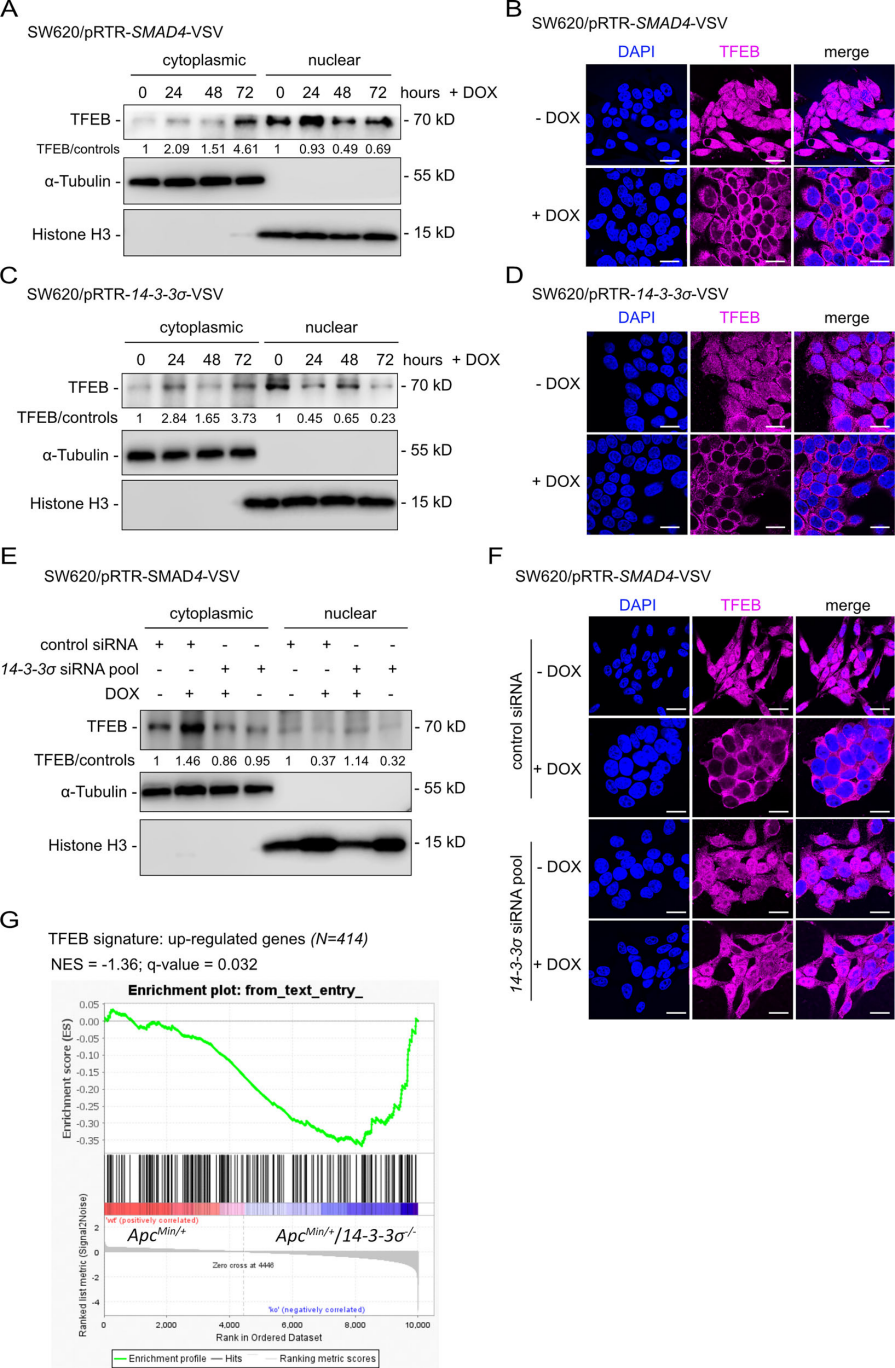
Cytoplasmic sequestration of TFEB by SMAD4 is mediated by 14-3-3σ. **A**, **C** Western blot analysis of TFEB content in the cytoplasm and nucleus after DOX treatment for the indicated periods. **B** and **D** Immunofluorescence analysis of TFEB after DOX treatment for 48 h. Nuclear DNA was stained with DAPI. Scale bar: 20 μm. **E** and **F** SW620 cells were transfected with the indicated siRNAs for 24 h and then treated with DOX for 48 h as indicated. **E** Western blot analysis of TFEB in the cytoplasm and nucleus. **F** TFEB was then analyzed by indirect immunofluorescence. Nuclear DNA was stained with DAPI. Scale bar: 20 μm. **G** GSEA analysis of mRNAs up-regulated by TFEB (*N* = 414) in intestinal tissues from *Apc*^Min/+^ and *Apc*^Min/+^/*14-3-3σ*^*−/−*^.

The transcription factor TFEB plays a central role in autophagy regulation [27]. The 14-3-3α/β/γ proteins are known to bind TFEB upon its phosphorylation by mTORC1 and subsequently inhibit TFEB by sequestration in the cytoplasm [29, 36, 37]. Therefore, we determined whether the SMAD4/14-3-3σ axis affects autophagy via regulating TFEB. Indeed, ectopic SMAD4 resulted in increased levels of TFEB in the cytoplasm while nuclear TFEB decreased (Fig. 7A, B). Also, ectopic expression of 14-3-3σ resulted in cytoplasmic sequestration of TFEB (Fig. 7C, D). After treatment with the nuclear export inhibitor leptomycin B, TFEB predominantly localized to the nucleus despite SMAD4 or 14-3-3σ activation, implying that SMAD4- or 14-3-3σ-induced TFEB nuclear-cytoplasmic translocation depends on nuclear export (Fig. S6A, B). Importantly, knockdown of *14-3-3σ* abolished the SMAD4-induced cytoplasmic retention of TFEB, indicating that SMAD4 induced cytoplasmic localization of TFEB is mediated by the SMAD4-induced 14-3-3σ protein (Fig. 7E, F). Furthermore, gene set enrichment analysis (GSEA) revealed an enrichment of mRNAs up-regulated by TFEB (*N* = 414) in intestinal adenomas from *14-3-3σ-*deficient mice (Fig. 7G). These findings indicate that inactivation of *14-3-3σ* resulted in up-regulation of TFEB target genes, providing evidence that 14-3-3σ acts as a repressor of TFEB transcriptional activity in vivo.

### Mechanisms and effects of 14-3-3σ-mediated sequestration of TFEB

**Fig. 8:**
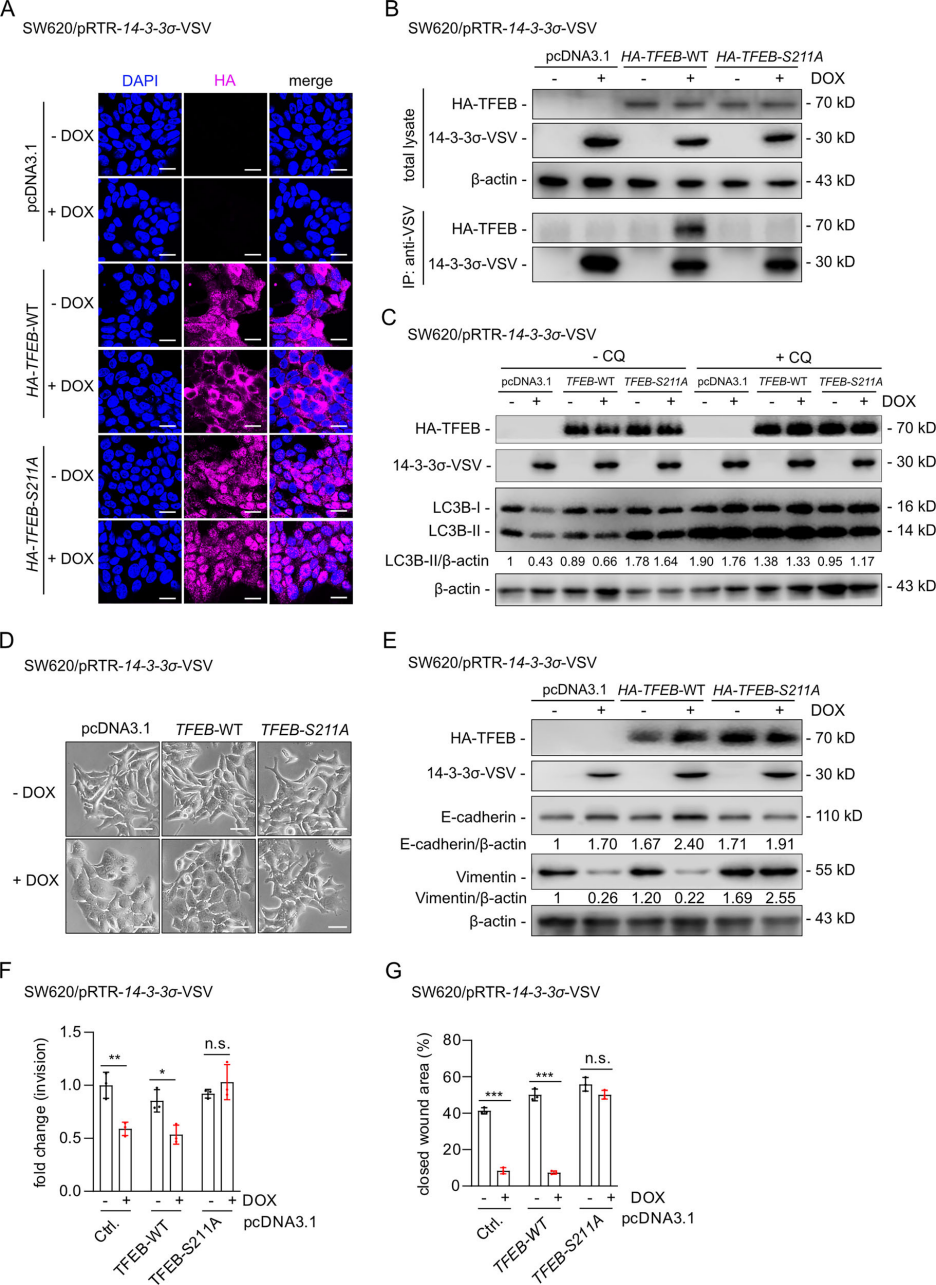
14-3-3σ sequesters S211-phosphorylated TFEB in the cytoplasm, thereby modulating autophagy and MET. **A** Cells were transiently transfected with the indicated plasmids following 24 h of treatment with or without DOX. After an additional 48 h, HA-tag expression was detected by indirect immunofluorescence analysis. Nuclear DNA was stained with DAPI. Scale bar: 20 μm. **B** Cells were treated with or without DOX for 24 h and then transfected with the indicated plasmids. Following an additional 48 h, cell lysates were subjected to immunoprecipitation (IP) with VSV-specific antibodies. After separation by gel electrophoresis, Western blotting analysis was used to detect the co-precipitated HA-tagged TFEB. “total lysates” indicate the expression of the proteins prior to immunoprecipitation. β-actin was used as a loading control. **C** Cells were transfected as in **A**, except that 20 μM of chloroquine was added 4 h before harvesting the cells. Immunoblotting was performed to assess the expression of the indicated protein. **D** After 24 h of treatment with or without DOX, cells were transfected with the indicated plasmids and then treated for another 48 h before analysis by phase-contrast microscopy. Scale bar: 50 μm. **E** Immunoblot assays analyze the indicated proteins after the treatments described in **D**. **F** Boyden chamber analysis was used to quantify cell invasion after cells were treated as described in **D**. **G** Quantification of wound healing after treatments as described in **D**. In **F**+**G** mean values ± SD are shown (*n = 3*). *p < 0.05, **p < 0.01, ***p < 0.001. n.s. no significance.

To investigate how 14-3-3σ sequesters TFEB in the cytoplasm, we transiently co-transfected plasmids encoding 14-3-3σ with either HA-tagged, wild-type or S211A mutant TFEB expressing plasmids, respectively. Ectopic expression of 14-3-3σ retained wild-type TFEB in the cytoplasm, whereas the mutant S211A/TFEB protein primarily localized in the nucleus even in the presence of ectopic 14-3-3σ (Fig. 8A). Correspondingly, 14-3-3σ bound to wild-type TFEB but not to the S211A/TFEB mutant as determined by immune-precipitation analysis (Fig. 8B). Therefore, phosphorylation at position S211 is crucial for the binding of 14-3-3σ to TFEB. Concomitant ectopic expression of wild-type TFEB with 14-3-3σ decreased autophagy, whereas the S211A-TFEB mutant antagonized the inhibitory effect of 14-3-3σ (Fig. 8C). In addition, concomitant expression of 14-3-3σ and wild-type TFEB resulted in MET, whereas ectopic, mutant TFEB-S211A prevented 14-3-3σ-induced MET and resulted in a mesenchymal phenotype (Fig. 8D). The expression of MET markers changed accordingly (Fig. 8E). Moreover, concomitant ectopic expression of 14-3-3σ and TFEB-WT reduced cell invasion and migration, whereas ectopic expression of TFEB-S211A alleviated the effects of 14-3-3σ (Fig. 8F, G, Fig. S7A, B). These results demonstrate that SMAD4-induced 14-3-3σ inhibits autophagy, EMT, invasion, and migration by direct sequestration of TFEB.

## Discussion

**Fig. 9:**
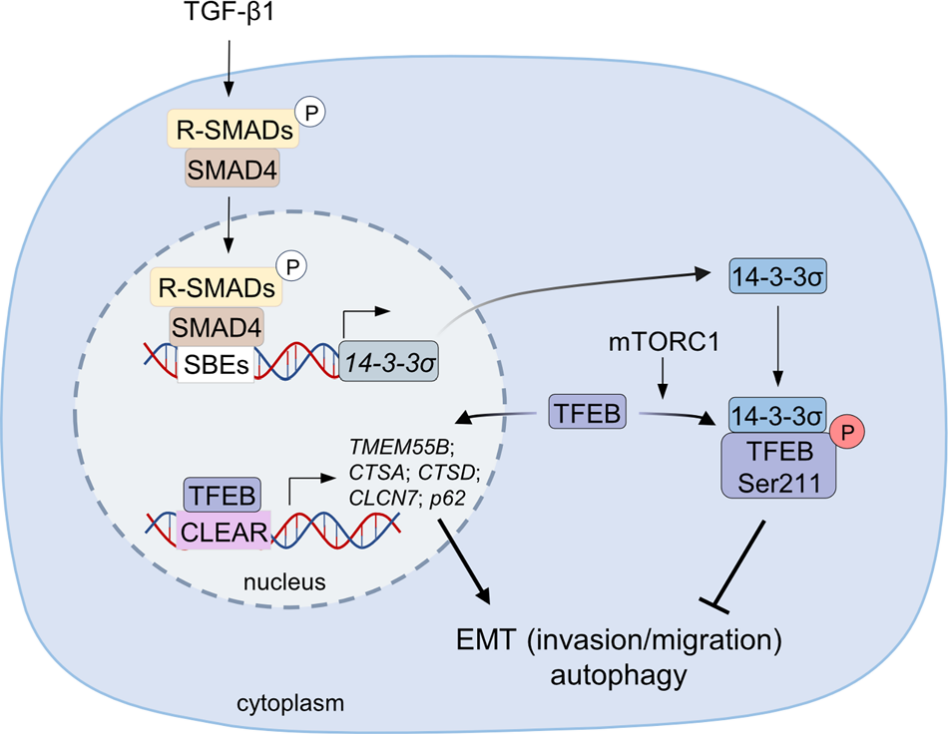
Schematic model of the TGF-β/SMAD4/14-3-3σ/TFEB signaling pathway. The scheme summarizes the findings of this study. Activation of SMAD4 by TGF-β results in its nuclear translocation and binding of SBEs within the *14-3-3σ* promoter and transcriptional activation of *14-3-3σ*. The resulting increase of cytoplasmic 14-3-3σ protein levels promotes sequestration of the transcription factor TFEB via binding to the phosphorylated Ser211 motif within TFEB. Therefore, the TFEB-/CLEAR-motif mediated transcription, which mediates autophagy and EMT, is inhibited, resulting in reduced invasion and migration, and presumably decreased survival of CRC cells. SBE SMAD-binding elements, CLEAR coordinated lysosomal and autophagic regulation.

Here we show that SMAD4 directly induces expression of the *14-3-3σ* gene in human and murine cells (see Fig. 9 for a summarizing scheme). The resulting increase in 14-3-3σ protein mediates down-stream effects of SMAD4, such as the induction of MET and inhibition of autophagy via sequestration of TFEB. These effects may ultimately contribute to SMAD4-mediated tumor suppression. For example, an increase in MET inhibits migration and invasion, and thereby prevents metastatic spread of cancer cells. Inhibition of autophagy may lower the threshold for apoptosis and prevent the survival of disseminated cancer cells. During therapy, a decrease of autophagy in tumor cells may increase their chemosensitivity. However, during tumor progression mutational inactivation of *SMAD4* and/or down-regulation of *14-3-3σ* may lead to activation of TFEB, which may have the opposite effect, and promote EMT and autophagy. Therefore, the molecular connections identified here are clinically relevant and may be exploited for therapeutic purposes in the future.

Here, we showed that the SMAD4-induced 14-3-3σ facilitates the re-localization of β-catenin to the membrane which is known to decrease the transcriptional activity β-catenin/TCF complexes [38]. Given that hyperactivation of Wnt/APC/β-catenin pathway constitutes a major hallmark of CRC [39, 40], the loss of SMAD4 during CRC progression may therefore contribute to further activation of the transcriptional activity of β-catenin/TCF through down-regulation of *14-3-3σ.* In addition, the SMAD4/14-3-3σ connection may also affect the PI3K/AKT, MAPK and Hippo/YAP signaling pathways, which have been shown to be down-stream of 14-3-3σ previously [41–43], and are involved in CRC tumorigenesis, therapeutic resistance and cellular plasticity.

Phosphorylation of TFEB at S211 by mTORC1 was shown to facilitate binding of the 14-3-3 family members 14-3-3α/β/γ next to a nuclear localization signal within the TFEB protein [29], thereby blocking nuclear translocation and preventing TFEB-driven expression of autophagy-related genes [44, 45]. Canonical SMAD signaling has been reported to precede mTORC1 activation in response to TGF-β1 stimulation [46]. Here, we show that 14-3-3σ specifically interacts with wild-type TFEB but not with the TFEB-S211A mutant, implying that phosphorylation of Ser211 is necessary for this interaction. mTORC1 is activated by multiple nutrient- and growth-related inputs, including amino acids via Rag GTPases [47], PI3K/AKT signaling [48], and cellular energy status sensed through AMPK-dependent regulation [49]. Since TFEB-induced autophagy has been implicated in promoting cancer progression through lysosomal biogenesis, EMT induction, cell cycle progression, and promotion of invasion in multiple cancer types [50–52], the TGF-β/SMAD4/14-3-3σ/TFEB axis may be a conserved tumor suppressive pathway in multiple types of cancers.

Consistent with the induction of MET by ectopic 14-3-3σ described here, deletion of the *14-3-3σ* has been shown to promote EMT [53]. EMT is associated with enhanced autophagy, which allows cell-survival under conditions of stress [54]. In addition, autophagy was shown to induce EMT and anoikis resistance, and thereby enhance metastatic potential [54–56]. Recent studies indicate that TFEB activates Wnt/β-catenin signaling, thereby promoting EMT and enhancing the migratory and invasive capacities of gastric cancer cells [57]. Here, we show that cytoplasmic sequestration of TFEB by 14-3-3σ inhibits both MET and autophagy. It remains to be determined whether these processes are independently regulated in this context or whether they are linked. Since this study relies on in vitro and ex vivo models for functional analyses, future studies should extend these findings to animal models in order to validate the effects in vivo. Taken together, the TGF-β/SMAD4/14-3-3σ/TFEB axis described here facilitates the coordinated control of autophagy and EMT. This may ultimately contribute to suppression of mCRC. On the contrary, inactivation of its components may promote CRC progression.

## Materials and methods

### Generation and handling of mice

*Smad4*^*fl/fl*^ mice (Stock No: 017462) were purchased from Jackson Laboratories. To generate mice with intestinal-specific knockout of *Smad4*, *Vil-CreERT2* mice were crossed with *Smad4*^*fl/fl*^ mice with a C57BL/6 background [58, 59]. All mice were housed in individually ventilated cages in the animal facility of the Biomedical Center (BMC) of the Ludwig-Maximilians-University (Munich, Germany). Mouse identification, healthy controls, and cage changes were performed under a laminar flow hood. Animal experimentation was approved by the local authorities. Genotyping primers are listed in Supplementary Table S1.

### Cell culture and treatments

The human patient-derived tumor organoids (PDTOs) used here were initially described and maintained as in [60]. Murine intestinal crypt isolation and organoid culture were performed as previously described in [59]. The human colorectal cancer cell lines SW480 and SW620 were cultured in McCoy’s 5A medium (Invitrogen, Carlsbad, CA, USA) supplemented with 10% fetal bovine serum (FBS) (Invitrogen) and 1% penicillin/streptomycin (Gibco, Thermo Fisher Scientific). Mouse colorectal cancer cells CT26 were cultured in RPMI1640 medium (σ-Aldrich, Gibco Life Technologies) with 10% FBS and 1% penicillin/streptomycin. All cells were cultured in an incubator with 20% O_2_, 5% CO_2_ and 37 °C. Doxycycline (DOX) (σ, St Louis, MO) was dissolved in water (100 μg/mL stock). The final concentration of DOX used for treatment was 100 ng/mL. To maintain stable cell pools containing the pRTR-vector plasmid [30], selection pressure was applied using puromycin at a final concentration of 8 μg/mL. Medium containing puromycin was replaced every 48 h to ensure continuous selection of transfected cells. FlexiTube GeneSolution GS2810 siRNA pools specific for *14-3-3σ* (containing 4 different *14-3-3σ-specific* siRNAs) and control siRNA pools were purchased from Qiagen (Hilden, Germany).

### RNA isolation and real-time polymerase chain reaction (qPCR) analysis

Total RNA was isolated from cultured cells according to the manufacturer’s instructions of the High Pure RNA Isolation Kit (Roche). cDNA was generated using the Verso cDNA Kit (Thermo Scientific), and mRNA was analyzed by qPCR using Fast Green Master Mix (Applied Biosystems) and LightCycler 480 II (Roche Diagnostics). For human genes, gene expression was normalized to *GAPDH* or *β-actin* using the 2^-ΔΔCt^ method [61], while murine gene expression was normalized to *Cyclophilin* or mouse *β-actin*. The primers used for qPCR are listed in Supplementary Table S2.

### Analysis of expression from public databases

Transcriptomic datasets were obtained from the NCBI Gene Expression Omnibus (GEO) using the search term “TGF-β” combined with relevant cell or tissue terms. The inclusion criteria for each dataset were either publicly available raw data or publicly available processed expression matrices with sufficient annotation or with appropriate sample information. Normalized expression values (RMA) and RPKM values were used from microarray and RNA-seq datasets, respectively. From each dataset, we extracted the expression values of *14-3-3σ* mRNA to compare fold changes between TGF-β-treated and control groups. The final dataset with the fold changes of *14-3-3σ* was plotted as bar plots with controls set to 1. Gene Set Enrichment Analysis (GSEA) was performed using the GSEA software provided by the Broad Institute. First, public GEO gene expression profiling datasets of cell lines/tissues with ectopic TFEB expression were analyzed to generate a TFEB signature of 414 genes, that were consistently up-regulated by TFEB. Next, the enrichment of the TFEB signature in the previously published RNA-seq dataset from *Apc*^*Min/+*^ and *Apc*^*Min/+*^*/14-3-3σ*^*−/−*^ mice [25] were analyzed. From GSEA, we obtained normalized enrichment scores (NES), FDR adjusted q-values, and generated enrichment plots showing the distributions of TFEB target genes.

### Chromatin immunoprecipitation

Cells were subjected to chromatin immunoprecipitation according to the instructions in the iDeal ChIP-qPCR kit (Diagenode, Belgium). Briefly, cells were cross-linked with 11% formaldehyde in fixation buffer for approximately 10 min at room temperature, followed by the addition of glycine at 1:10 volume ratio with gentle shaking for 5 min at room temperature. After washing with PBS, samples were collected and lysed using the buffers provided in the kit (Buffer iL1B and iL2). Chromatin was fragmented into 200–500 bp fragments by sonication using shearing buffer iS1b, and shearing efficiency was verified by agarose gel electrophoresis. Equal amounts of sheared chromatin were incubated with antibodies against SMAD4 or negative control IgG overnight at 4 °C, and the immune complexes were captured using protein A-coated magnetic beads. Wash with appropriate wash buffers iW1 to iW4 to eliminate non-specific binding. Reverse crosslinking was performed with complete DIB buffer containing proteinase K. Separated DNA was then quantified by qPCR to determine enrichment at selected promoters or regulatory regions. The relative amount of immunoprecipitated DNA compared to input DNA for the control regions was calculated using the following formula: %recovery = 2^[(Ct_input_ – 6.64) - Ct_sample_] * 100%. Positive and negative control areas were included to assess the specificity and efficiency of the ChIP assay. Sequences of qChIP primers and antibodies are provided in Supplementary Tables S3.

### Modified Boyden-chamber assay

To measure the invasion ability, Matrigel (Corning) and serum-free culture medium were mixed at a ratio of 1:6 and added to the culture chamber. The membrane was incubated at 37 °C for 3 h to form a coating. 1 × 10^5^ cells were seeded in the upper chamber of serum-free culture medium (8.0 μM pore size filter, Corning), and the culture medium containing 10% fetal bovine serum as a chemo-attractant was seeded in the lower chamber. After 48 h, the invasive cells located on the lower surface of the chamber membrane were fixed with methanol for 30 min, stained with 0.5% crystal violet for 20 min, and then photographed and counted under a microscope. The fold change of invasive cells was calculated and normalized by comparing it with the corresponding control group.

### Wound healing assay

The cells were seeded and cultured in culture-inserts (80241; IBIDI, Martinsried, Germany) until cells became confluent and formed a cell monolayer without intercellular spaces. The cells were treated with 10 μg/mL Mitomycin C (M4287, Sigma-Aldrich, Germany) for 2 h, and then the culture-inserts were removed. After washing twice with HBSS, fresh culture medium was added. The cells were photographed immediately with a phase contrast microscope and observed and photographed with a phase contrast microscope 24 h later.

### Protein immunoprecipitation analysis

Protein immunoprecipitation analysis was performed according to the instructions of the Classic Magnetic IP/Co-IP Kit (Thermo Scientific, catalog number: 88804). Cells were seeded in 100 × 100 mm culture dishes. After the designated treatments, each dish was supplemented with 1 mL of IP Lysis/Wash Buffer and incubated on ice 5 min. The lysates were then collected and centrifuged at 4 °C for 10 min. The supernatant was transferred to a new tube, and protein concentration was determined using a BCA assay. 600 μg of total protein was incubated with 2 μg of the specific antibody. The antibody-lysate mixture was adjusted to a final volume of 500 μL with IP Lysis/Wash Buffer and rotated overnight (12–16 h) at 4 °C. 25 μL (0.25 mg) of Protein A/G magnetic beads were pre-washed with IP Lysis/Wash Buffer and added to each antibody-lysates mixture. Samples were rotated at room temperature for 1 h to allow immune complex binding. Beads were then collected with a magnetic rack and washed three times with 500 μL of IP/Wash Buffer, followed by one rinse with ultrapure water. Bound proteins were eluted with 100 μL of elution buffer at room temperature for 10 min with gentle mixing, and the eluates were subsequently denatured at 95 °C before immunoblot analysis. The list of antibodies is shown in Table S4.

### Western blot analysis

Cells were washed with HBSS and lysed with RIPA lysis buffer containing mini protease inhibitor (Roche) and PhosSTOP Phosphatase Inhibitor Cocktail Tablets (Roche). The cell lysate was sonicated for 5 s and then centrifuged at 13,000 rpm for 20 min at 4 °C to collect the supernatant containing proteins, which were then quantified according to the instructions of the Pierce^TM^ BCA protein Assay Kit (Thermo Fisher Scientific). Protein samples were separated by electrophoresis on 10% or 12% SDS-PAGE gels and transferred to PVDF membranes (Millipore). The ECL (Millipore) system was used and images were obtained with a LI-COR Odyssey FC Imaging System (Bad Homburg, Germany). Western blots were quantified using Image J software. A list of antibodies is provided in Table S4.

### Colony formation assay

Cells were seeded in 6-well plates and treated as indicated. The treated cells were seeded into 12-well plates at a density of 1000 cells/well, and colony formation was measured after 2 weeks. After methanol fixation and crystal violet staining, colonies were recorded with a digital camera (Nikon, Japan) and counted using Image J software.

### In situ hybridization analysis (ISH)

The analysis was performed as described previously [62]. In brief, formaldehyde-fixed tissue samples were placed on glass slides. Cell membranes were permeabilized by 30 μg/mL proteinase K treatment. *14-3-3σ*-specific in situ RNA probes were generated as described previously [25]. The probes were added to the samples, hybridized at 65 °C, and washed to remove unbound probes. The signals were detected using the BCIP/NBT Liquid Substrate System (Sigma), and the signal locations were observed under a microscope to show the spatial distribution of the target nucleic acid. The slides were scanned using a Vectra® Polaris^TM^ Imaging System.

### Immunohistochemical analysis

Mouse small intestine tissues were fixed with formalin. Paraffin-embedded sections (5 μm) were deparaffinized in xylene and rehydrated through a graded ethanol series. Antigen retrieval was performed by microwaving the slides in Target Retrieval Solution Citrate (pH 6.0, Dako) at 750 W for 25 min. After cooling to room temperature, endogenous peroxidase activity was blocked with 3% hydrogen peroxide for 10 min. Blocking was then carried out following the protocol of the ImmPRESS HRP Horse Anti-Rabbit (or Anti-Mouse) IgG Polymer Kit (Vector Laboratories). Primary antibodies were diluted in Dako Antibody Diluent with Background Reducing Components, applied to slides and incubated overnight at 4 °C. After washing with PBS, the sections were treated with an HRP-conjugated antibody for 1 h at room temperature. The Dako Liquid DAB Substrate Chromogen System was then used for visualization and sections were counterstained with hematoxylin (Vector). Cover-slips were applied using Roti®-Histokitt II (Roth). Slides were scanned using a Vectra® Polaris^TM^ Imaging System. Data analysis was conducted using Phenochart version 2.0.0 software. A list of antibodies is provided in Table S4.

### Immunofluorescence analysis by confocal laser-scanning microscopy

Cells were cultured on round glass slides in 12-well plates and then fixed in 4% paraformaldehyde for 15 min, permeabilized with 0.2% Triton X-100 for 10 min, and blocked with 5% BSA in PBS for 1 h at the room temperature. Next, primary antibodies were incubated at 4 °C for 12 to 16 h in a humidified chamber, washed three times with PBS-Tween 20, and then incubated with appropriate secondary antibodies for 1 h at room temperature. Cell chromatin was stained with DAPI (Roche) for 5 min. After final washes, coverslips were mounted with ProLong Gold anti-fluorescent dye (Invitrogen). Images were acquired using a confocal microscope (LSM 700, Zeiss) with 63x Plan-Apochromat oil immersion lens and ZEN 2009 software (Zeiss). Puncta quantification of LC3B was performed using ImageJ software. For each condition, at least three microscopic fields encompassing a total of no fewer than 200 cells were analyzed. Antibodies are listed in Table S4.

### Statistical analysis

Statistical analysis was performed using GraphPad Prism 10 software (USA). Statistical differences between the two groups were calculated using Student’s t-test (two-tailed; unpaired). Asterisks generally indicate: *p < 0.05, **p < 0.01, and ***p < 0.001, n.s. no significant.

## Supplementary information

Supplemental Figures and Tables

aj-checklist reporting

## Data Availability

All data, analytic methods, and study materials will be made available to other researchers upon reasonable request.
