# Supplementary material for: TGF-β/SMAD4/14-3-3σ/TFEB axis promotes mesenchymal-epithelial transition and inhibits autophagy in colorectal cancer
Source: Cell Death Dis. 2026 Apr 21;17(1):397. doi: 10.1038/s41419-026-08733-x (PMC13100165; doi:10.1038/s41419-026-08733-x)
Supplement: Supplementary file 1 — Supplemental Figures and Tables [file 41419_2026_8733_MOESM1_ESM.pdf]

Supplementary Material

TGF-β/SMAD4/14-3-3σ/TFEB axis promotes mesenchymal-epithelial transition and inhibits autophagy in colorectal cancer

Xiaoyan Chen, Markus Winter, Matjaz Rokavec, Janine König and Heiko Hermeking

Inventory of Supplementary Material:

Supplementary Figure 1
Related to Figure 1
14-3-3σ is a direct target gene of SMAD4 in human CRC cells

Supplementary Figure 2
Related to Figure 2
SMAD4 is required for 14-3-3σ expression in the large intestinal epithelium in vivo

Supplementary Figure 3
Related to Figure 3
SMAD4 induced MET inhibits invasion and wound healing

Supplementary Figure 4
Related to Figure 5
SMAD4 induced MET is mediated by 14-3-3σ

Supplementary Figure 5
Related to Figure 6
14-3-3σ overexpression leads to a decrease in the number of LC3B puncta

Supplementary Figure 6
Related to Figure 7
TFEB cytoplasmic localization by SMAD4 or 14-3-3σ requires nuclear export

Supplementary Figure 7
Related to Figure 8
14-3-3σ regulates cell invasion and metastasis by binding to phosphorylated TFEB at S211

Supplementary Figure 8
Original Western blots (Uncropped membranes)

Supplementary Table S1
Oligonucleotides used for mouse genotyping

Supplementary Table S2
Oligonucleotides used for qPCR

Supplementary Table S3
Oligonucleotides used for qChIP

Supplementary Table S4
List of antibodies

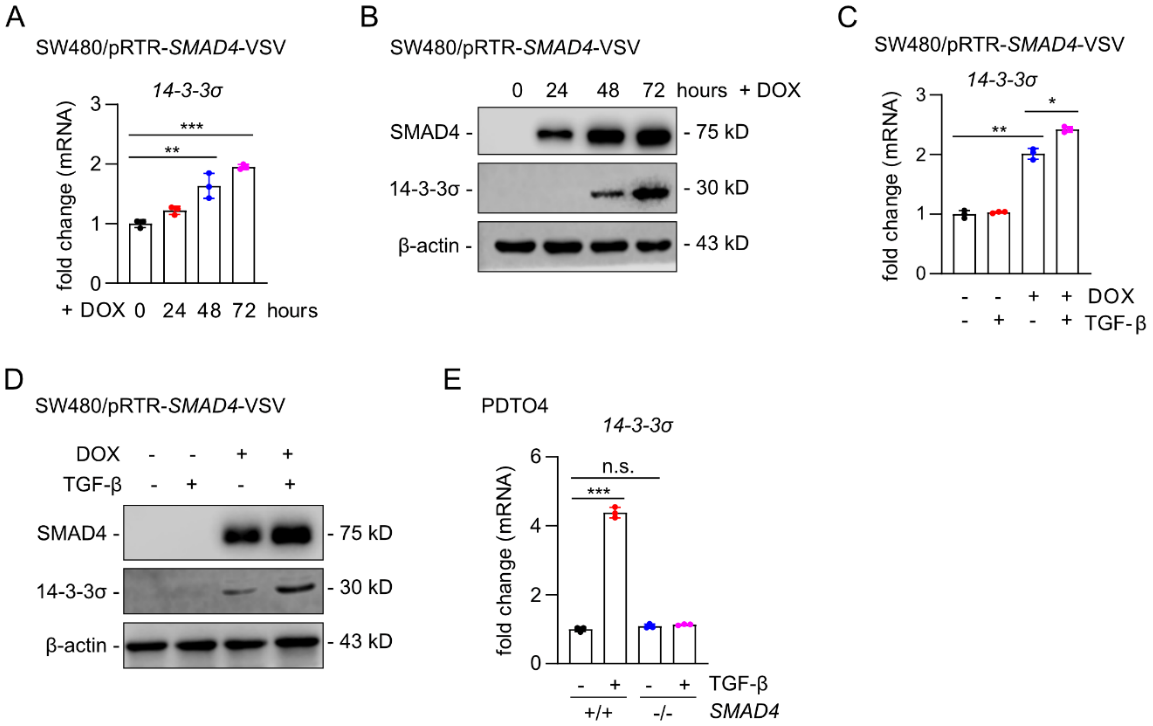

Figure S1: ***14-3-3σ* is a direct target gene of SMAD4 in human CRC cells**

(A) qPCR analysis of *14-3-3σ* expression after SMAD4 induction with DOX for the indicated periods. (B) Western blot analysis of 14-3-3σ in SW480 cells treated as in (A). (C) qPCR analysis of *14-3-3σ* expression after treatment with DOX and/or 20 ng/mL recombinant TGF-β1 for 48 hours. (D) Western blot analysis of 14-3-3σ in SW480 cells treated as in (C). (E) qPCR analysis of *14-3-3σ* expression in *SMAD4* wild-type and *SMAD4* knockout PDTO4 after treatment with/without 20 ng/mL recombinant TGF-β1 for 72 hours. Results are presented as the mean ± SD (n = 3) for panel A, C, E and I with *: $p < 0.05$, **: $p < 0.01$, ***: $p < 0.001$. n.s. no significance.

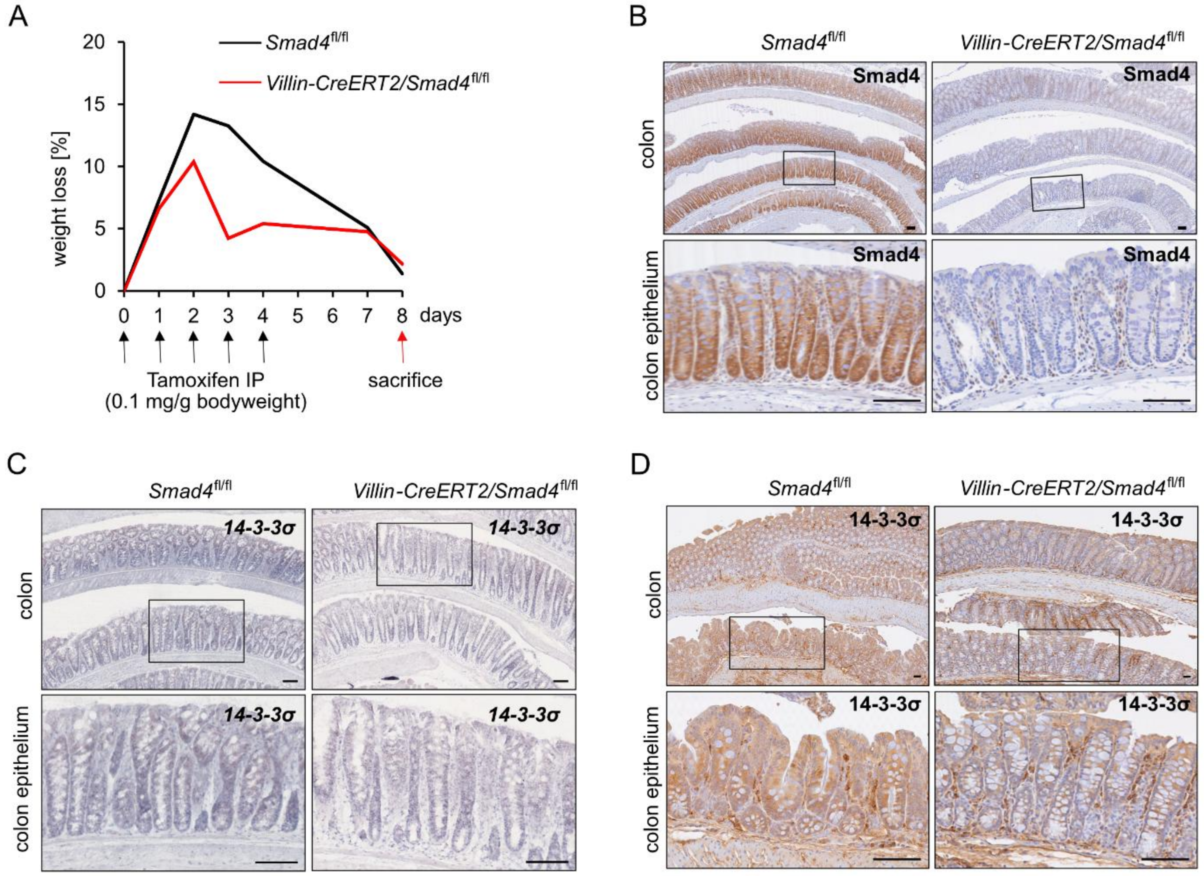

Figure S2: SMAD4 is required for 14-3-3σ expression in the large intestinal epithelium in vivo

(A) 8-week-old mice of both genotypes were injected intraperitoneally with tamoxifen at a dose of 0.1 mg/g of mouse body weight once daily. The mice were weighed daily and sacrificed after four days. (B) Immunohistochemical detection of Smad4 in the colon of 8-week-old mice of indicated genotypes. n=3 mice per genotype. Scale bar represents 100 μm. (C) *14-3-3σ* mRNA was detected by ISH in the colon of 8 weeks old mice of the indicated genotypes. n=3 mice per genotype. Scale bar represents 100 μm. (D) Immunohistochemical detection of 14-3-3σ in the colon of 8 weeks old mice of the indicated genotype. n=3 mice per genotype. Scale bar represents 100 μm.

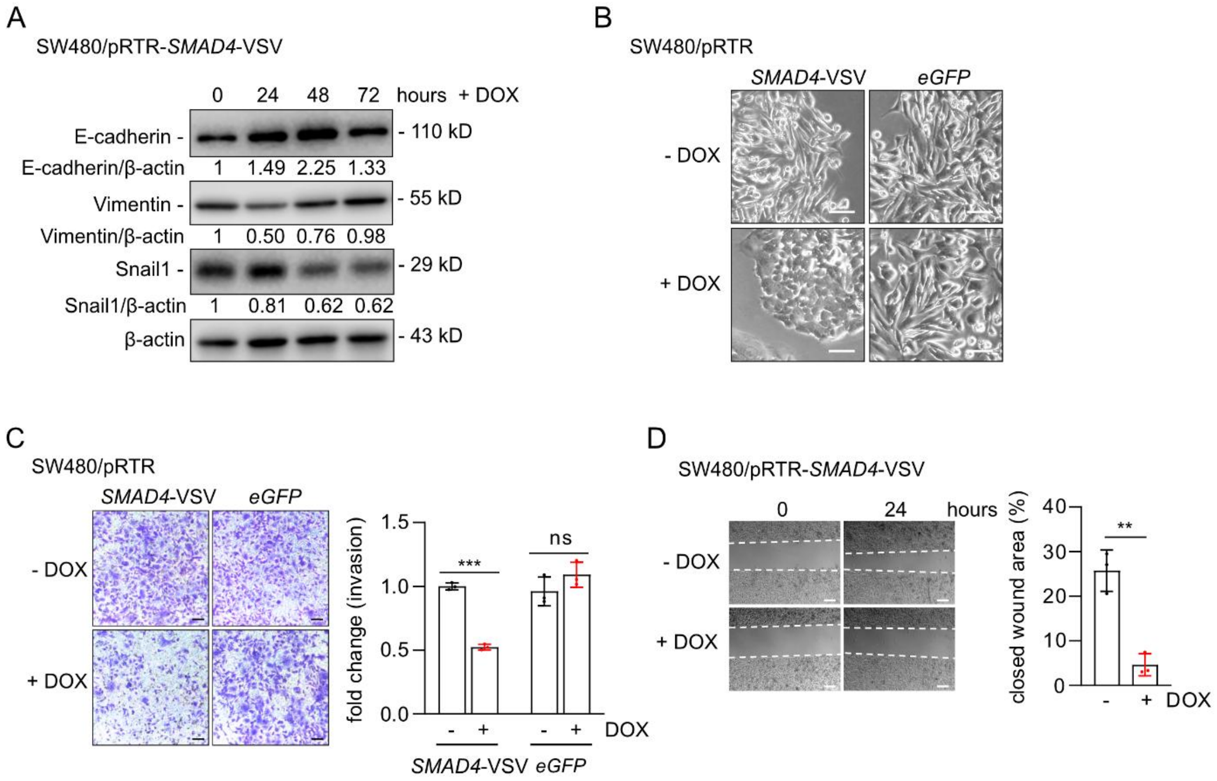

Figure S3: SMAD4 induced MET inhibits invasion and wound healing

(A) Western blot analysis of indicated proteins after SMAD4 induction by DOX for the indicated periods in SW480 cells. (B) Representative phase contrast images show the morphology of SW480 cells with or without the addition of DOX. eGFP activated by the addition of DOX served as a control. (C) Invasion was measured using a modified Boyden chamber assay. After 48 hours of DOX treatment in SW480 cells, the number of cells that invaded through the Matrigel was counted by crystal violet staining. (D) Wound healing assay measured the width of the wound after 48 hours of treatment with or without DOX in SW480 cells, and observed the change in width after 24 hours of scratching (left). The results represent the mean (%) of wound healing (right) in SW480 cells. Scale bar: 50 μm. Results are presented as the mean ± SD (n = 3) for panel C and D with **: $p < 0.01$, ***: $p < 0.001$. n.s. no significance.

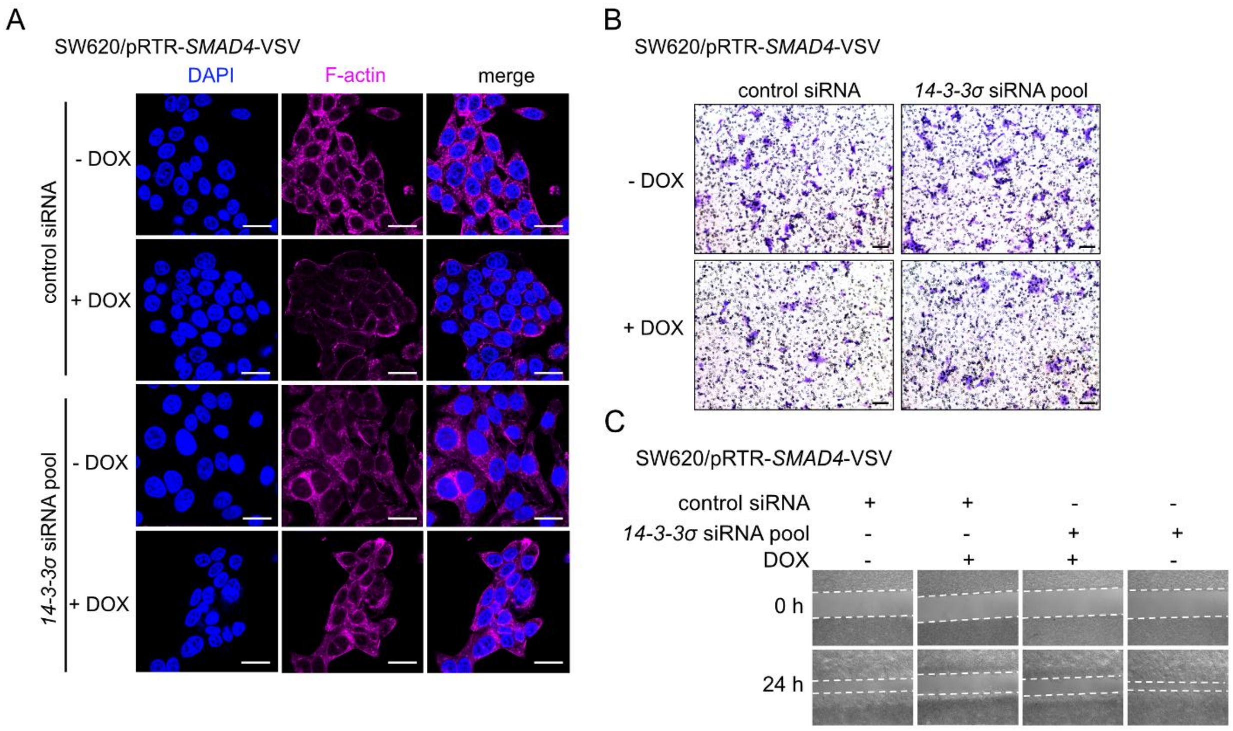

Figure S4: SMAD4 induced MET is mediated by 14-3-3σ

(A) After SMAD4 was activated with DOX and simultaneously transfected with the indicated siRNAs in SW620 cells for 72 hours, the cells were subjected to immunofluorescence staining for F-actin. Nuclear DNA was stained with DAPI. Scale bar: 20 μm. (B) Cell invasion ability was measured using a modified Boyden chamber assay. SW620 cells were treated as in (A), and the number of cells that invaded through Matrigel was counted using crystal violet staining. Scale bar: 50 μm. (C) The wound healing assay measured the wound width of SW620 cells after being treated as shown in (A), and observed the changes in wound width 24 hours after wounding. Scale bar: 50 μm.

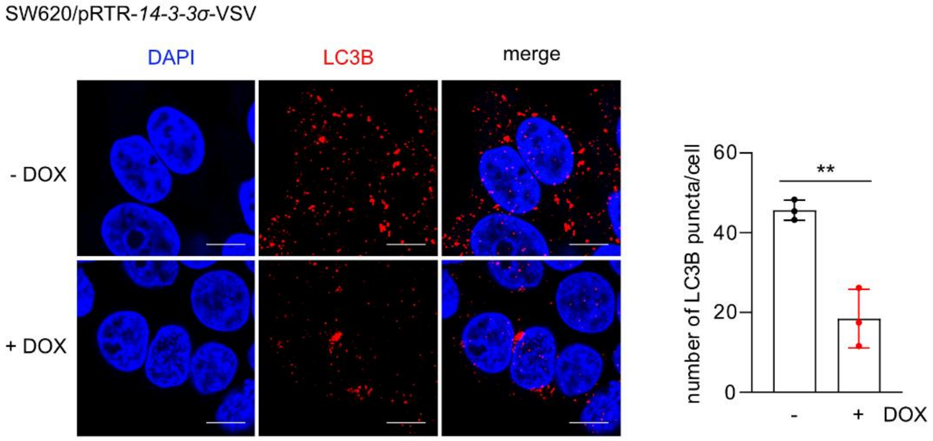

Figure S5: 14-3-3σ overexpression leads to a decrease in the number of LC3B puncta

Immunofluorescence microscopy was used to detect LC3B in SW620 cells that treated with DOX for another 48 hours. Nuclear DNA was stained with DAPI. Scale bar: 20 μm. The bar graphs are based on the results of immunofluorescence counting.

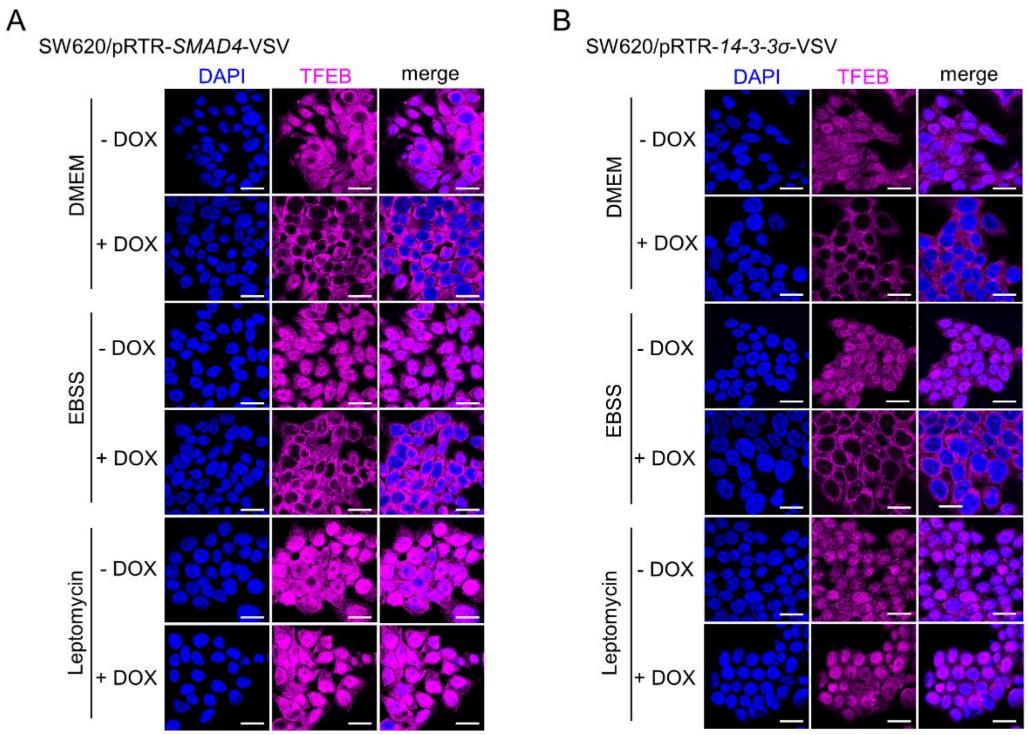

Figure S6: TFEB cytoplasmic localization by SMAD4 or 14-3-3σ requires nuclear export

(A) and (B) After 24 hours of treatment with or without DOX, SW620 cells were treated with DMEM, EBSS (serum-free, starvation treatment), or Leptomycin (2.5ng/mL, a nuclear export inhibitor) for an additional 24 hours as indicated. DOX treatment was performed for a total of 48 hours.

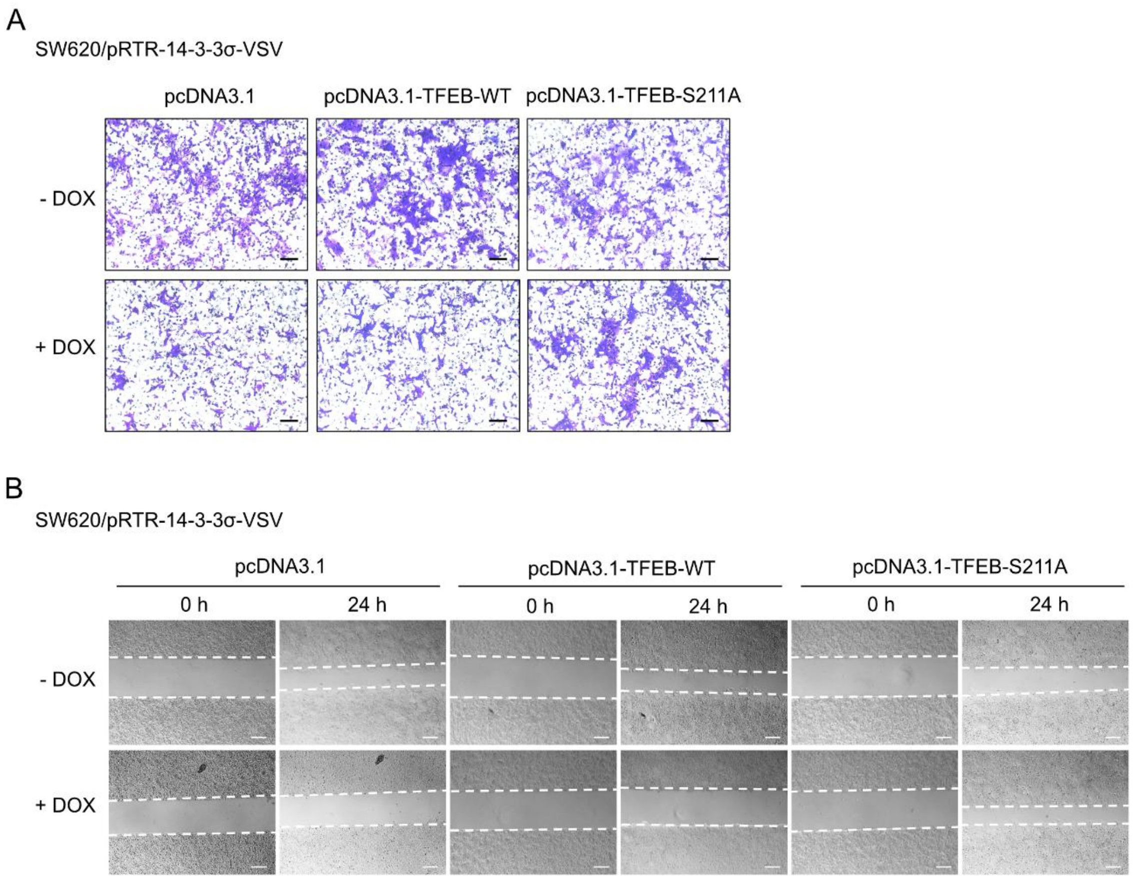

Figure S7: 14-3-3σ regulates cell invasion and metastasis by binding to phosphorylated TFEB at S211

(A) Cell invasion ability was measured using a modified Boyden chamber assay. Cells were transiently transfected with the indicated plasmids following 24 hours of treatment with or without DOX. After an additional 48 hours, the number of cells that invaded through Matrigel was counted using crystal violet staining. Scale bar: 50 μm. (B) The wound healing assay measured the wound width of SW620 cells after being treated as shown in (A), and observed the changes in wound width 24 hours after wounding. Scale bar: 50 μm.

Figure S8: Original blots

Uncropped gel for Figure 1B

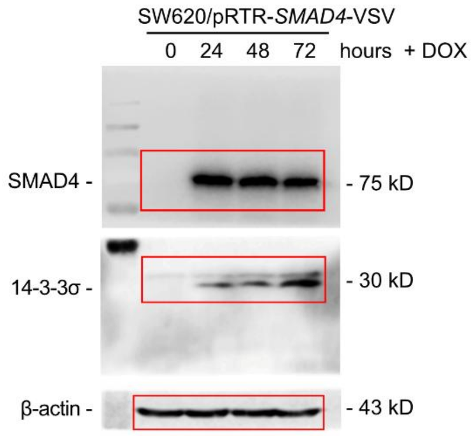

Uncropped gel for Figure 1D

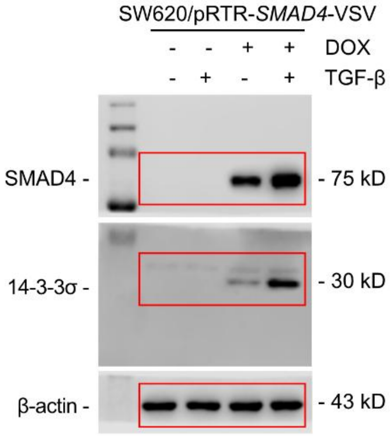

Uncropped gel for Figure 1F

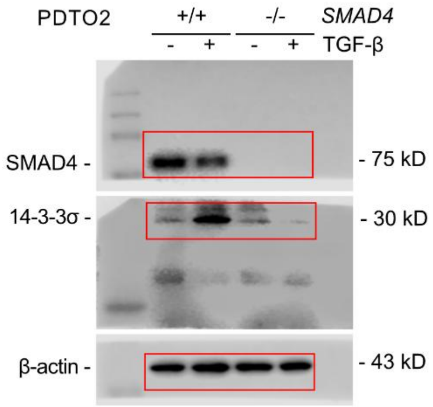

Uncropped gel for Figure 2B

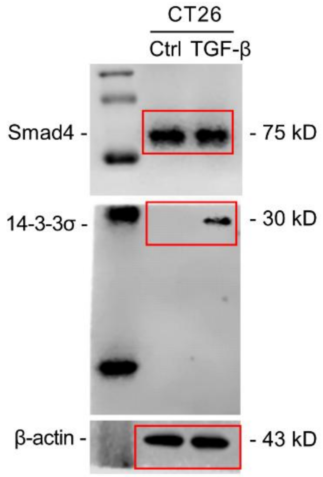

Uncropped gel of Figure 2F

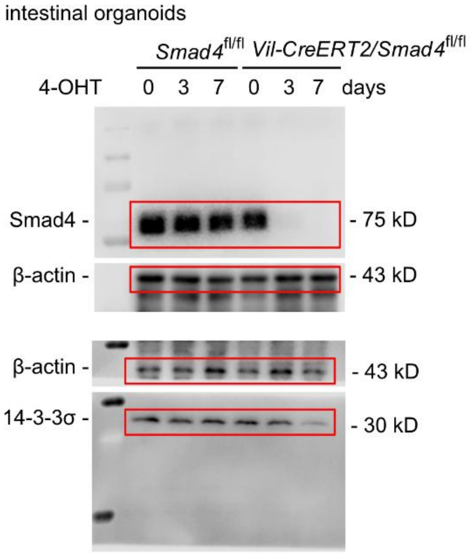

Uncropped gel for Figure 3A left panel

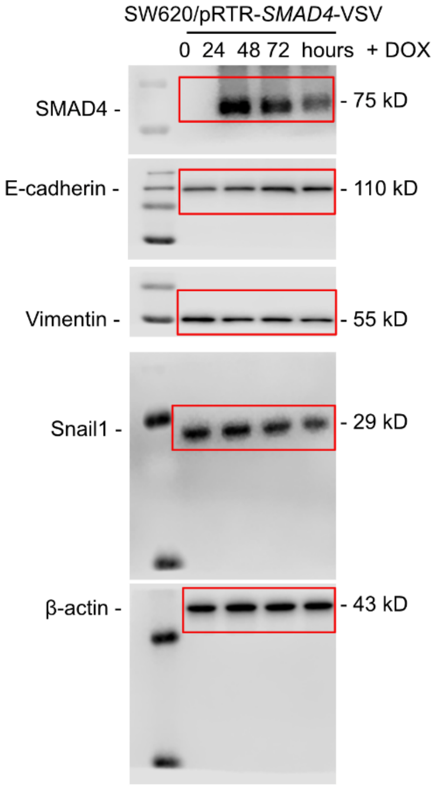

Uncropped gel for Figure 3A right panel

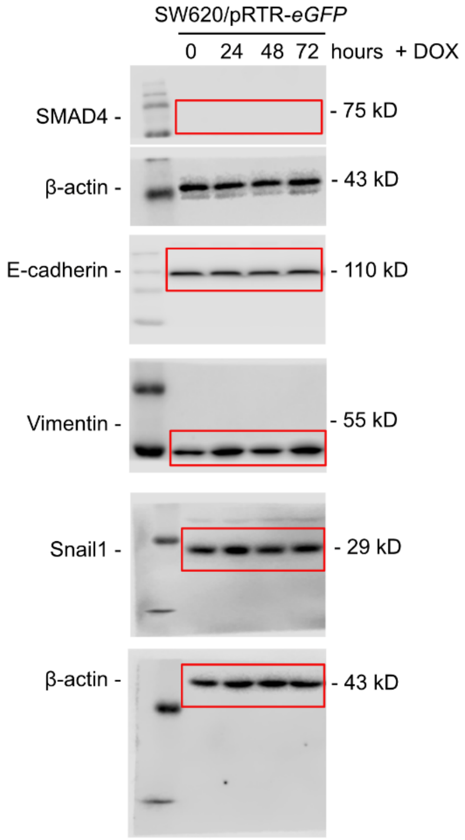

Uncropped gel for Figure 4A left panel

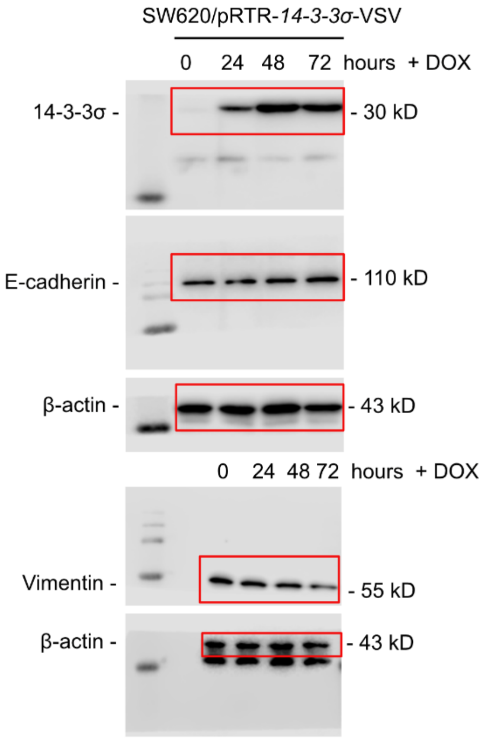

Uncropped gel for Figure 4A right panel

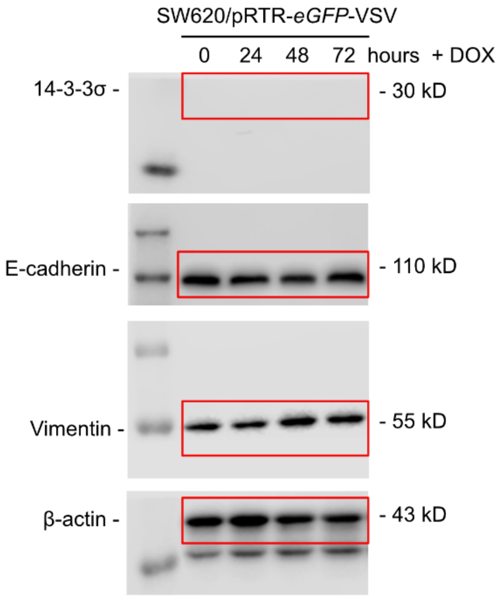

Uncropped gel for Figure 5A

SW620/pRTR-*SMAD4*-VSV

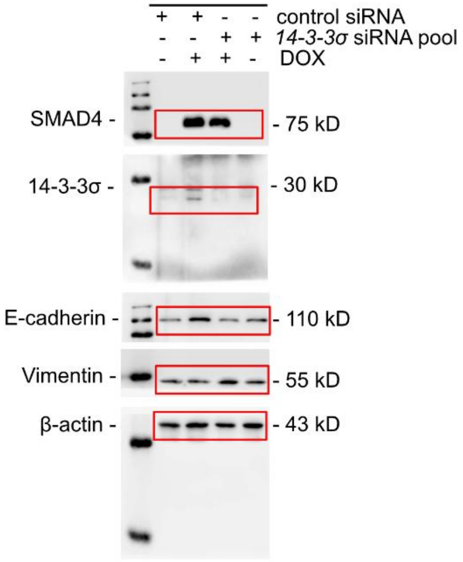

Uncropped gel for Figure 6A

SW620/pRTR-*SMAD4*-VSV

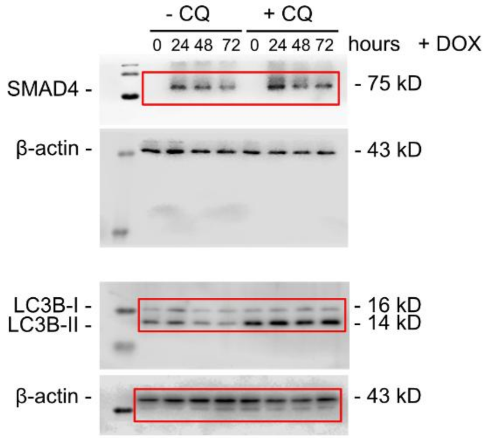

Uncropped gel for Figure 6B

SW620/pRTR-*14-3-3σ*-VSV

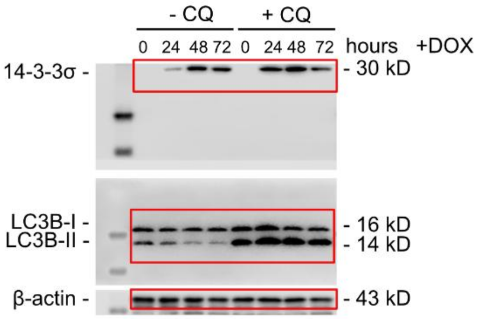

Uncropped gel for Figure 6C

SW620/pRTR-*SMAD4*-VSV

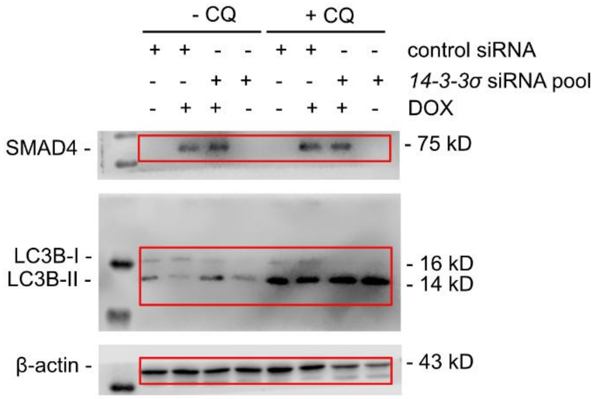

Uncropped gel for Figure 7A

SW620/pRTR-*SMAD4*-VSV

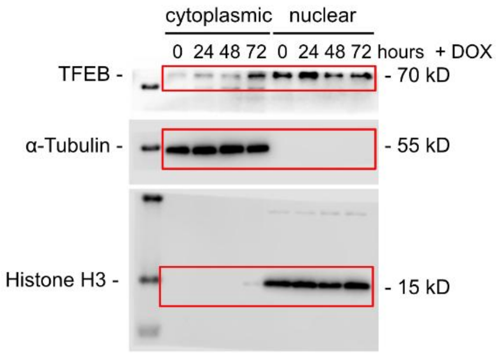

Uncropped gel for Figure 7C

SW620/pRTR-*14-3-3σ*-VSV

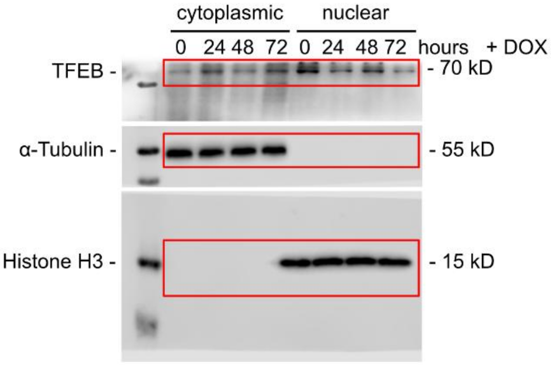

Uncropped gel for Figure 7E

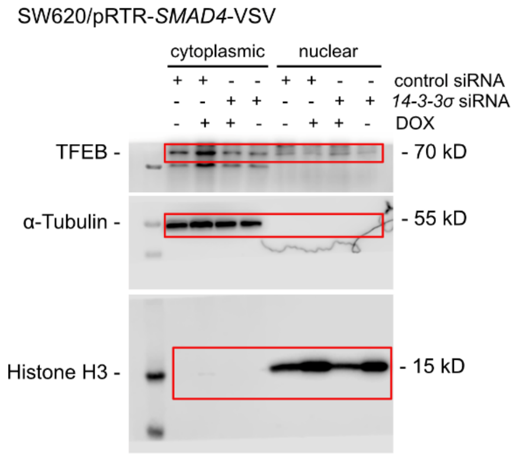

Uncropped gel for Figure 8B

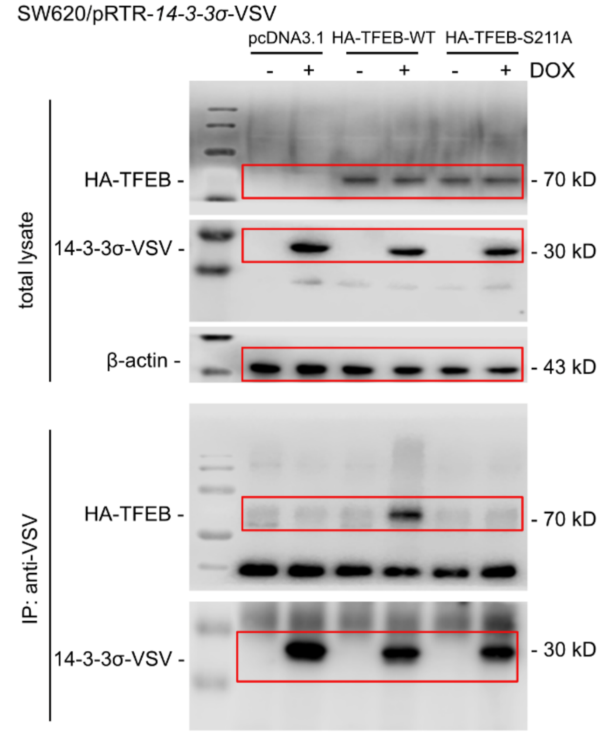

Uncropped gel for Figure 8C

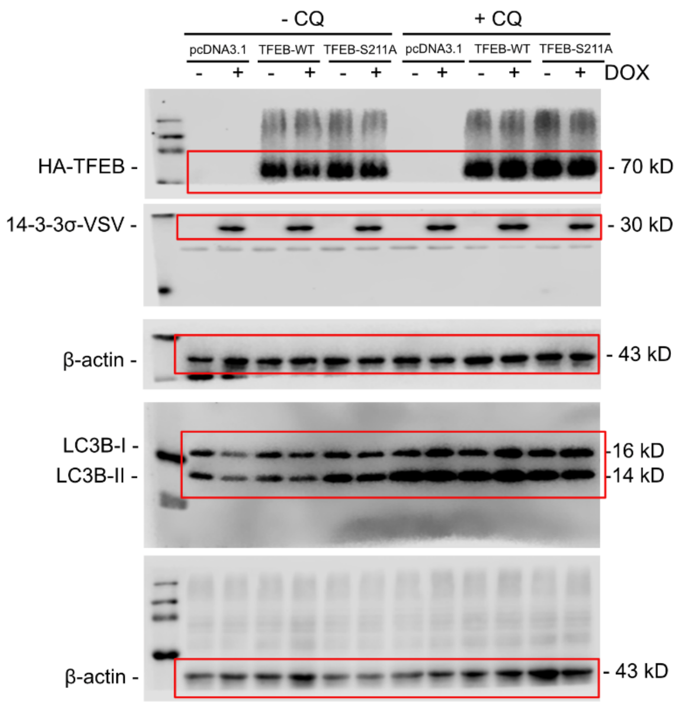

Uncropped gel for Figure 8E

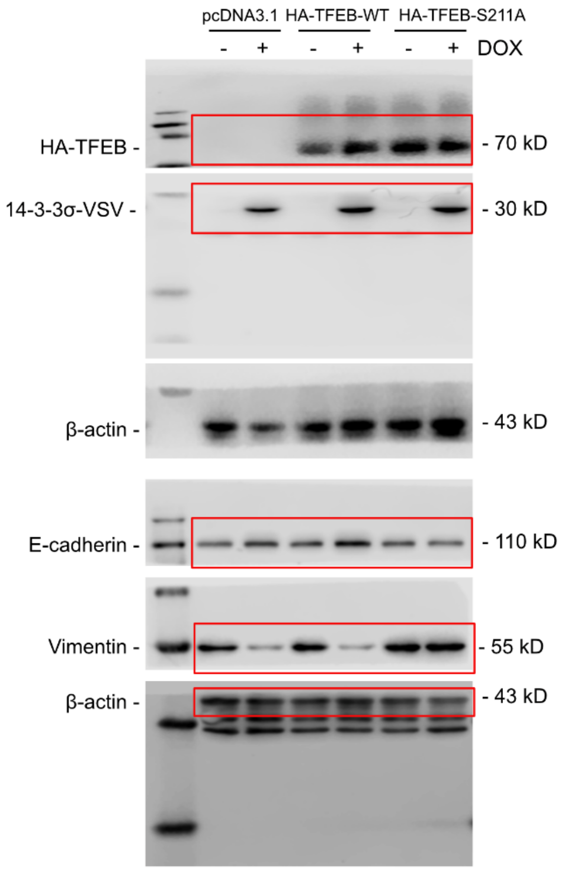

Uncropped gel for Figure S1B

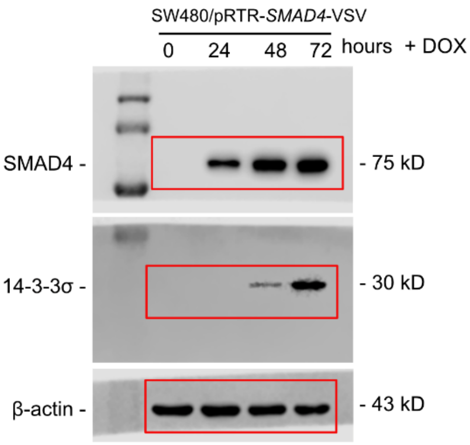

Uncropped gel for Figure S1D

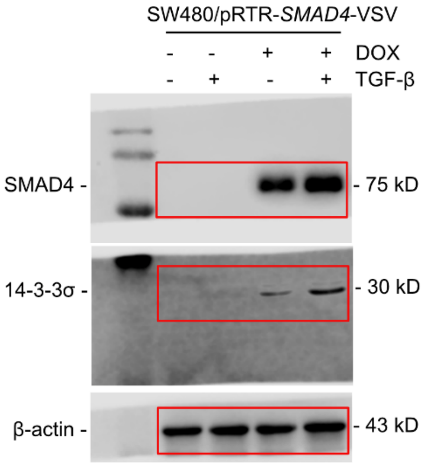

Uncropped gel of Figure S3D

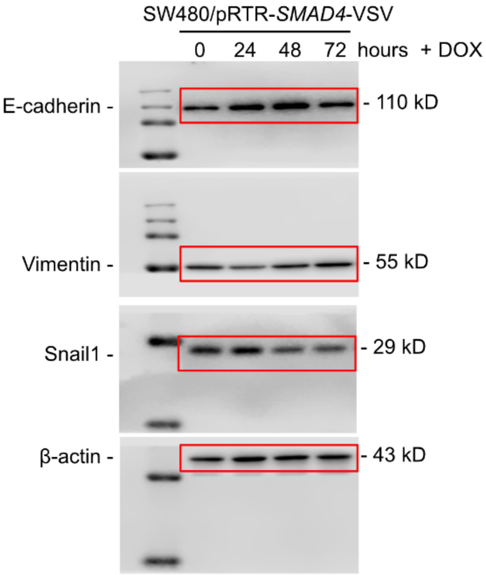

Table S1. Oligonucleotides used for mouse genotyping.

Name	Sequence (5’-3’)
Vil-CreERT2-for	CCTGGAAAATGCTTCTGTCCG
Vil-CreERT2-rev	CAGGGTGTTATAAGCAATCCC
Gabra (Internal control)-for	CAATGGTAGGCTCACTCTGGGAGATGATA
Gabra (Internal control)-rev	AACACACACTGGCAGGACTGGCTAGG
Smad4 $^{fl/fl}$-for	TAAGAGCCACAGGGTCAAGC
Smad4 $^{fl/fl}$-rev	TTCCAGGAAAAACAGGGCTA

Table S2. Oligonucleotides used for qPCR.

Name	Sequence (5’-3’)
Mouse-*Cyclophilin*-for	ATGGTCAACCCCACCGTGT
Mouse-*Cyclophilin*-rev	TTCTGCTGTCTTTGGAACTTTGTC
Mouse-*β-actin*-for	CTAAGGCCAACCGTGAAAAG
Mouse-*β-actin*-rev	ACCAGAGGCATACAGGGACA
Mouse-*Smad4*-for	CAGCCATAGTGAAGGACTGTTGC
Mouse-*Smad4*-rev	CCTACTTCCAGTCCAGGTGGTA
Mouse-*14-3-3σ*-for	CATGAAGAGCGCCGTGGAAAAG
Mouse-*14-3-3σ*-rev	CTCTTCTGCTCGATGCTGGACA
Human-~~*GADPH*~~-for	TGTTGCCATCAATGACCCCTT
Human-~~*GADPH*~~-rev	CTCCACGACGTACTCAGCG
Human-*TFEB*-for	CCTGGAGATGACCAACAAGCAG
Human-*TFEB*-rev	TAGGCAGCTCCTGCTTCACCAC
Human-*Smad4*-for	CTACCAGCACTGCCAACTTTCC
Human-*Smad4*-rev	CCTGATGCTATCTGCAACAGTCC
Human-*14-3-3σ*-for	AAGATGAAGGGTGACTACTA
Human-*14-3-3σ*-rev	GACCGGGCTGAGTCAATGA
Human-*TMEM55B*-for	GTTCGATGCCCCTGTAACTGTC
Human-*TMEM55B*-for	CCCAGGTTGATGATTCTTTTGC
Human-*CTSA*-for	CAGGCTTTGGTCTTCTCTCCA
Human-*CTSA*-rev	TCACGCATTCCAGGTCTTTG
Human-*CTSD*-for	AACTGCTGGACATCGCTTGCT
Human-*CTSD*-for	CATTCTTCACGTAGGTGCTGGA
Human-*CLCN7*-for	TGATCTCCACGTTCACCCTGA
Human-*CLCN7*-rev	TCTCCGAGTCAAACCTTCCGA
Human-*p62*-for	CCCTACAGATGCCAGAATCCG
Human-*p62*-rev	GCCTTCATCAGAGAAGCCCAT
Human-TFEB-for	CCTGGAGATGACCAACAAGCAG
Human-TFEB-rev	TAGGCAGCTCCTGCTTCACCAC

Table S3. Oligonucleotides used for qChIP.

Gene	Sequence (5'-3')
Human-*14-3-3σ* (site A)-for	TATAAATTGGAGAGAATAAGA
Human-*14-3-3σ* (site A)-rev	GACAGGGTCTCACCATGTTGC
Human-*14-3-3σ* (site B)-for	CTTGGACTAAAGGCTAACACAAGAGTGAA
Human-*14-3-3σ* (site B)-rev	TCATGCCATTCTCCTACCTCAGCC
Human-*Smad7*-for	CTGCCTAGGGCATTCATT
Human-*Smad7*-rev	CCAGCTTGGGTATATGTTTC
Human-*16q22*-for	CTACTCACTTATCCATCCAGGCTAC
Human-*16q22*-rev	ATTTCACACACTCAGACATCACAG
Mouse-*Smad7*-for	AAACCCGATCTGTTGTTTGC
Mouse-*Smad7*-rev	GGCCGTCTAGACACCCTGT
Mouse-*14-3-3σ*-for	CTCTGCGTGCTGACCTCATTC
Mouse-*14-3-3σ*-rev	AAGGCAATGCTGTTCGAAGTA
Mouse-*AchR*-for	AGTGCCCCCTGCTGTCAGT
Mouse-*AchR*-rev	CCCTTTCCTGGTGCCAAGA

Table S4. List of antibodies.

Primary antibodies

Epitope	Catalog No.	Company	Use	Dilution	Source
α-Tubulin	#T-9026	Sigma-Aldrich	WB	1:1000	mouse
β-actin	#A2066	Sigma-Aldrich	WB	1:1000	rabbit
14-3-3σ	# sc-166473	Santa Cruz	WB	1:1000	mouse
14-3-3σ	#Ab14123	Abcam	IHC	1:200	mouse
Smad4	# sc-7966	Santa Cruz	WB; IHC	1:1000; 1:200	mouse
E-cadherin	# 33-4000	Invitrogen	WB/IF	1:1000; 1:200	mouse
Vimentin	# 5741	Cell Signaling Technology	WB	1:1000	rabbit
Snail	# 3879	Cell Signaling Technology	WB	1:1000	rabbit
β-catenin	# 8480	Cell Signaling Technology	IF	1:100	rabbit
LC3B	# 2775	Cell Signaling Technology	WB/IF	1:1000; 1:200	rabbit
TFEB	# Ab270604	Abcam	WB/IF	1:1000; 1:100	rabbit
Histone H3	# 9715	Cell Signaling Technology	WB	1:1000	rabbit
Anti-HA	#26183	Invitrogen	WB	1:1000	mouse
Anti-VSV	# V4888	Sigma-Aldrich	WB/IP	1:5000 2μg/IP	rabbit

F-actin	# A12379	Thermo Fisher	IF	1:50	Alexa Fluor® 488 conjugated

Secondary antibodies

Name	Catalog No.	Company	Use	Dilution	Source
Anti-mouse HRP	#W4021	Promega	WB	1:10,000	goat
Anti-rabbit HRP	#A0545	Sigma-Aldrich	WB	1:10,000	goat
Goat anti-mouse 555	#A21422	Thermo Fisher	IF	1:1000	goat
Goat anti-rabbit 647	#A32733	Thermo Fisher	IF	1:1000	goat
